# Water oxidation couples to electrocatalytic hydrogenation of carbonyl compounds and unsaturated carbon–carbon bonds by nickel

**DOI:** 10.1038/s41598-022-23777-7

**Published:** 2022-11-19

**Authors:** Leila Behrouzi, Zahra Zand, Mobina Fotuhi, Babak Kaboudin, Mohammad Mahdi Najafpour

**Affiliations:** 1 Department of Chemistry, Institute for Advanced Studies in Basic Sciences (IASBS), Zanjan, 45137-66731 Iran; 2 Center of Climate Change and Global Warming, Institute for Advanced Studies in Basic Sciences (IASBS), Zanjan, 45137-66731 Iran; 3 Research Center for Basic Sciences & Modern Technologies (RBST), Institute for Advanced Studies in Basic Sciences (IASBS), Zanjan, 45137-66731 Iran

**Keywords:** Electrocatalysis, Heterogeneous catalysis

## Abstract

Artificial photosynthesis, an umbrella term, is a chemical process that biomimetics natural photosynthesis. In natural photosynthesis, electrons from the water-oxidation reaction are used for carbon dioxide reduction. Herein, we report the reducion of aldehydes and ketones to corresponding alcohols in a simple undivided cell. This reaction utilized inexpensive nickel foam electrodes (1 cm^2^) and LiClO_4_ (0.05 M) as a commercially accessible electrolyte in an aqueous medium. Under electrochemical conditions, a series of alcohols (21 examples) produces high selectivity in good yields (up to 100%). Usage the current method, 10 mmol (1060 mg) of benzaldehyde is also successfully reduced to benzyl alcohol (757 mg, 70% isolated yield) without any by‑products. This route to alcohols matched several green chemistry principles: (a) atom economy owing to the use of H_2_O as the solvent and the source of hydrogen, (b) elimination of the homogeneous metal catalyst, (c) use of smooth reaction conditions, (d) waste inhibition due to low volumetric of by-products, and (e) application of safe EtOH co-solvent. Moreover, the ability of the system to operate with alkyne and alkene compounds enhanced the practical efficiency of this process.

## Introduction

**Figure 1:**
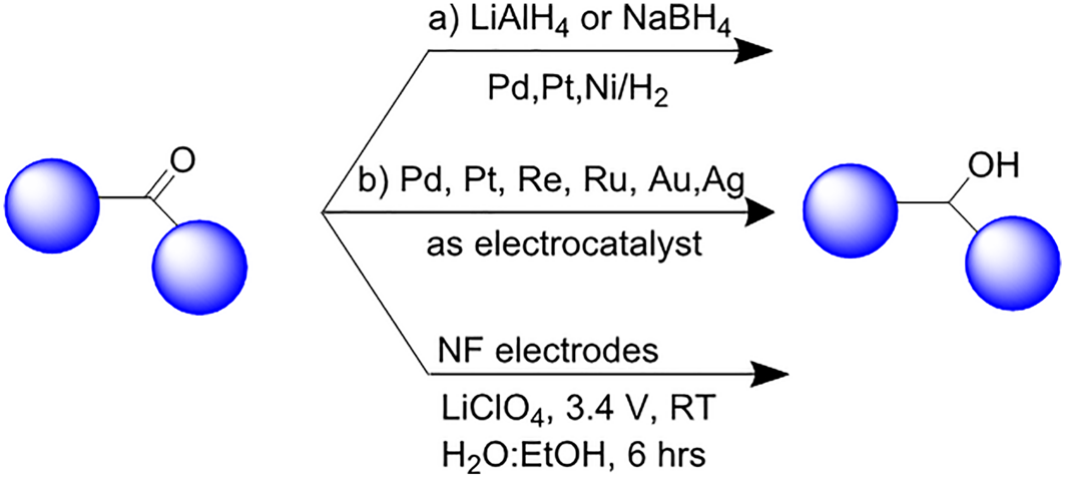
Hydrogenation strategies of carbonyl compounds. Strategy (**a**): classic chemical approach, Strategy (**b**): previous electrochemical methods, and Strategy (**c**): This work.

Alcohols find in pharmaceuticals, natural products, agrochemicals, dyes, and polymers^1–3^. The existing methods for carbonyl reduction frequently require metals (e.g. palladium, platinum, ruthenium, osmium, nickel and iron) or stoichiometric reductant reagents (e.g. NaBH_4_, LiAlH_4_ and H_2_) that commonly show limited substrate scope (Fig. 1a)^4,5^. These systems have demonstrated some drawbacks, such as: using precious, low abundance and toxic metals. On the other hand, hydrogen gas is very flammable and lacks compatibility with a diverse range of functional groups^6^. Also, the reductants are costly, and metal salts form as by-products during of reaction^7^.

In recent years, synthetic organic electrochemical protocols have received significant attention since electrochemistry proposes a mild and environmentally-friendly alternative to conventional chemical approaches for organic reactions^8–11^. To the best of our knowledge, the electrochemical conversion of carbonyl to corresponding alcohols has a short history of merely several decades, and most of the methodology leads to pinacol products^12–15^. Calvo et al. reported the electrochemical reduction of ketones to corresponding alcohols using the palladium wire as a working electrode^16^. Gutierrez et al. developed an electrochemical system using Pd, Rh and Pt catalysts. The resulting catalyst system was successfully applied to reduce butyraldehyde, furfural, acetophenone, and benzaldehyde^17^. Mechanistic studies of electrochemical hydrogenation of carbonyl compounds were conducted by Lilga’s group^18^. Lercher and Gutiérrez's group examined the carbon-supported Pd, Re, Pt and Ni catalysts for benzaldehyde reductio^19^. Cathodic reduction of 5-hydroxymethylfurfural was performed on the solid electrode in the presence and the absence of glucose by Koper's group^20^. A similar study in 2016 showed that the reduction of 5-hydroxymethylfurfural on solid electrodes in an acidic solution produced 2,5- Bis(hydroxymethyl) furan, 2,5- Bis(hydroxymethyl) tetrahydrofuran, and 2,5-dimethylfuran^21^. Li et al. pointed to mechanisms of electrochemical reduction of furfural on metallic Cu electrodes in acidic media. This mechanism may happen through either electrocatalytic hydrogenation or electro-reduction paths^22^. In a subsequent study, Attard’s group examined the reduction of ketopantolactone (KPL) and ethyl pyruvate (EP) on platinum electrodes^23,24^. Koper and co-workers reported the hydrogenation of acetophenone, 4-acetylpyridine, and acetone at platinum single-crystal electrodes (Fig. 1b)^25,26^.

Artificial photosynthesis is a chemical process that biomimetic natural photosynthesis. In the mentioned process, water and carbon dioxide are transformed into carbohydrates and oxygen using sunlight^27^. Indeed, in natural photosynthesis, electrons from the water-oxidation reaction are used for carbon dioxide reduction. Oxygen-evolution reaction (OER) by water-oxidation reaction is a fundamental electrode reaction owing to its essential applications in electrochemical energy apparatus such as regenerative fuel cells and rechargeable metal-air batteries^28–31^. Ru and Ir oxides have been employed in the OER reactions as catalysts, though low abundance and high cost have limited their widespread use^32^. Recently, increasing attempts have been made to design Ni, Co, Mn and Fe-based transition metal oxides or hydroxides that replaced Ru- and Ir-based OER catalysts^33-39^. Ni and Cu foams have been widely noticed as electrodes in OER reactions due to their 3D conductive frameworks and porous structures^40–43^. The high porosity and surface area of 3D nanoporous foams show increased conductivity compared to 2D planar electrodes^44^. For example, Li et al. thermally synthesized a 3D NiO/Ni foam as OER catalysis. The oxidation of nickel compounds under thermal conditions led to the NiO film^45^. Also, a simple one-step process for synthesizing NiO on the surface of Ni foam during electrochemical alcohol oxidation, without the need for an external thermal or nickel source, is reported in our previous article^46^.

While carbonyl compounds are reduced on solid-metal electrodes using expensive homogeneous and heterogeneous metals, we propose an alternative electrochemical methodology without requiring specialized reductants via low-cost OER electrons (Fig. 1c).

## Results and discussion

### Reduction of carbonyl and other functional groups

Changing several electrodes as anode and cathode seriously affected the reaction efficiency (Table 1). Running the reaction with aluminum, magnesium, iron, and titanium cathode produced 4%, 37%, 32% and 5% yields (entries 1–4, respectively). Testing of other anodes, such as titanium, did not improve the flow of the reaction, while nickel–iron foam showed an improvement process (entries 5 and 6). The iron foam anode improved by 95% product with high selectivity (entry 7). The highest yield was obtained using nickel foam as both the anode and cathode, while the formation of by-products was not observed (entry 8).

**Table 1:** Optimization of reaction conditions. For all of these reactions, < 10% maximum error is offered.


Entry	Electrode (Cathode/Anode)	Electrolyte	Solvent (5:1)	Yield (%)^ab^
1^c^	Mg/NF	LiClO_4_	H_2_O: EtOH	4^a^ (–)^b^
2	Al/NF	LiClO_4_	H_2_O: EtOH	37^a^ (24)^b^
3	Fe/NF	LiClO_4_	H_2_O: EtOH	32^a^ (20)^b^
4	Ti/NF	LiClO_4_	H_2_O:EtOH	5^a^ (–)^b^
5	NF/Ti	LiClO_4_	H_2_O:EtOH	trace^a^ (–)^b^
6	NF/ FeNF	LiClO_4_	H_2_O:EtOH	54^a^ (41)^b^
7	NF/FeF	LiClO_4_	H_2_O: EtOH	95^a^ (82)^b^
**8**	**NF/NF**	**LiClO**_**4**_	**H**_**2**_**O: EtOH**	**100**^a^ (92)^b^
9	NF/NF	NaCl	H_2_O: EtOH	trace^a^ (–)^b^
10	NF/NF	LiBr	H_2_O: EtOH	–
11	NF/NF	KIO_3_	H_2_O: EtOH	49^a^ (32)^b^
12	NF/NF	–	H_2_O: EtOH	–
13^d^	NF/NF	LiClO_4_	H_2_O: EtOH	59^a^ (45)^b^
14	NF/NF	LiClO_4_	H_2_O: CH_3_CN	100^a^ (92)^b^
15	NF/NF	LiClO_4_	EtOH	trace^a^ (–)^b^
16^e^	NF/NF	LiClO_4_	H_2_O: EtOH	25^a^ (–)^b^
17^f^	NF/NF	LiClO_4_	H_2_O: EtOH	16^a^ (9)^b^

Significant values are in bold.

^a^Product yields were determined by GC analysis. ^b^Isolated yield of products. ^c^H_2_O: milli-Q water. ^d^Decreasing the amount of electrolyte to 0.2 mmol. ^e^E = 2.3 V instead of E = 3.4 V. ^f^E = 1.3 V; all reactions performed under air atmosphere and surface of the electrodes was 1 cm^2^.

Replacing LiClO_4_ with other electrolytes such as NaCl, LiBr, and KIO_3_ led to a notable drop in the yield (entries 9–11). Unsurprisingly, no reaction occurred without electrolytes (entry 12). Moreover, by changing the electrolyte to 0.2 mmol, the amount of product decreased to 59% (entry 13). While CH_3_CN and ethanol were almost equally efficient, the latter choice was for its superior safety profile (entry 14). However, the reaction did not proceed when ethanol was used instead of a mixture of ethanol and H_2_O (entry 15). Changing the voltage to 2.3 V or 1.3 V decreased the yield of the reaction (entries 16 and 17).

So, the final optimized conditions for reducing benzaldehyde to benzyl alcohol are as follows: LiClO_4_ as the supporting electrolyte (0.5 mmol) in H_2_O: EtOH (6 mL) under Constant- potential conditions in an undivided cell.

With the optimized reaction conditions in hand, we probed a wide range of aldehydes and ketones to explore the ability and compatibility of this electrochemical hydroxylation reaction. As shown in Table 2, various benzaldehydes with different substituents offered the corresponding alcohols in moderate to excellent yields.

**Table 2:** Substrate scope of carbonyl compounds.


		
**2a**, 6 h, 100%^a^ (92%)^b^	**2b**, 20 h, 93%^a^ (85%)^b^	**2c**, 20 h, 75%^a^ (61%)^b^
[Ref]^54^ 90% yield	[Ref]^54^–	[Ref]^54^–
[Ref]^55^ 99% yield	[Ref]^55^–	[Ref]^55^–
		
**2d**, 20 h, 100%^a^ (90%)^b^	**2e**, 20 h, 33%^a^ (21%)^b^	**2f**, 6 h, 72%^a^ (59%)^b^
[Ref]^54,55^–	[Ref]^54,55^–	[Ref]^54,55^–
		
**2g,** 20 h, 100%^a^ (90%)^b^	**2h**, 24 h, 100%^a^ (92%)^b^	**2i**, 20 h, 54%^a^ (40%)^b^
[Ref]^54^–	[Ref]^54^–	[Ref]^54^ 94% yield
[Ref]^55^–	[Ref]^55^ 94% yield	[Ref]^55^ 95% yield
		
**2j**, 20 h, 44%^a^ (31%)^b^	**2k**, 22 h, 10%^a^ (trace)^b^	**2l**, 24 h, 100%^a^ (91%)^b^
[Ref]^54,55^–	[Ref]^54,55^–	[Ref]^54,55^–
		
**2m**, 22 h, 100%^a^ (91%)^b^	**2n**, 24 h, 16%^a^ (10%)^b^	**2o**, 22 h, 56%^a^ (43%)^b^
[Ref]^54,55^–	[Ref]^54,55^–	[Ref]^54,55^–
		
**2p**, 20 h, 100%^a^ (93%)^b^	**2q**, 23 h, 88%^a^ (71%)^b^	**2r**, 20 h, 7%^a^ (trace)^b^
[Ref]^54,55^–	[Ref]^54^ 93% yield	[Ref]^54,55^–
[Ref]^54,55^–	[Ref]^55^ 95% yield	[Ref]^54,55^–
		
**2s**, 23 h, trace^a^ (–)^b^	**2t**, 23 h, trace^a^ (–)^b^	
[Ref]^54,55^–	[Ref]^54,55^–	

^a^Product yields were determined by GC analysis.

^b^Isolated yield of products.

Simple benzaldehyde could furnish the desired benzyl alcohol in a good yield (2a). Fortunately, *o*-bromo, *o*-chloro, and *m*- chloro benzaldehyde worked well in this system and provided the products (2b, 2c, 2d) in 75–100% yields. Our results indicated that electron-withdrawing groups such as cyano, chloro, and bromo at the para position afforded 21%, 59%, and 90% isolated yields. Electron-donating groups like isopropyl, methyl, and methoxy at the para position were all converted in 30–100% yields (2 h–2j). The reduction of 2, 6-dichlorobenzaldehyde was more challenging than the 2, 4-dichlorobenzaldehyde due to its satirically hindered. 2, 4-Dimethylbenzaldehyde and 2, 4-dichlorobenzaldehyde provided the corresponding alcohols in 100% yield (2 l and 2 m). Notably, 2, 4-dimethoxybenzaldehyde produced the desired alcohol in a trace yield (2n). The 1-naphthaldehyde and 2-naphthaldehyde substrates provided the desired 2o and 2p in 45% and 57% yield, respectively. As examples of ketones, Acetophenone successfully transformed into 1-phenylethanol in 88% yield (2q), while the reduction of benzophenone failed (2r). No product was detected when cyclohexanone and hexanal were utilized as reaction substrates (2 s and 2t). In all cases, the yield obtained from this method has been very successful compared to the results of previous reports.

**Figure 2:**
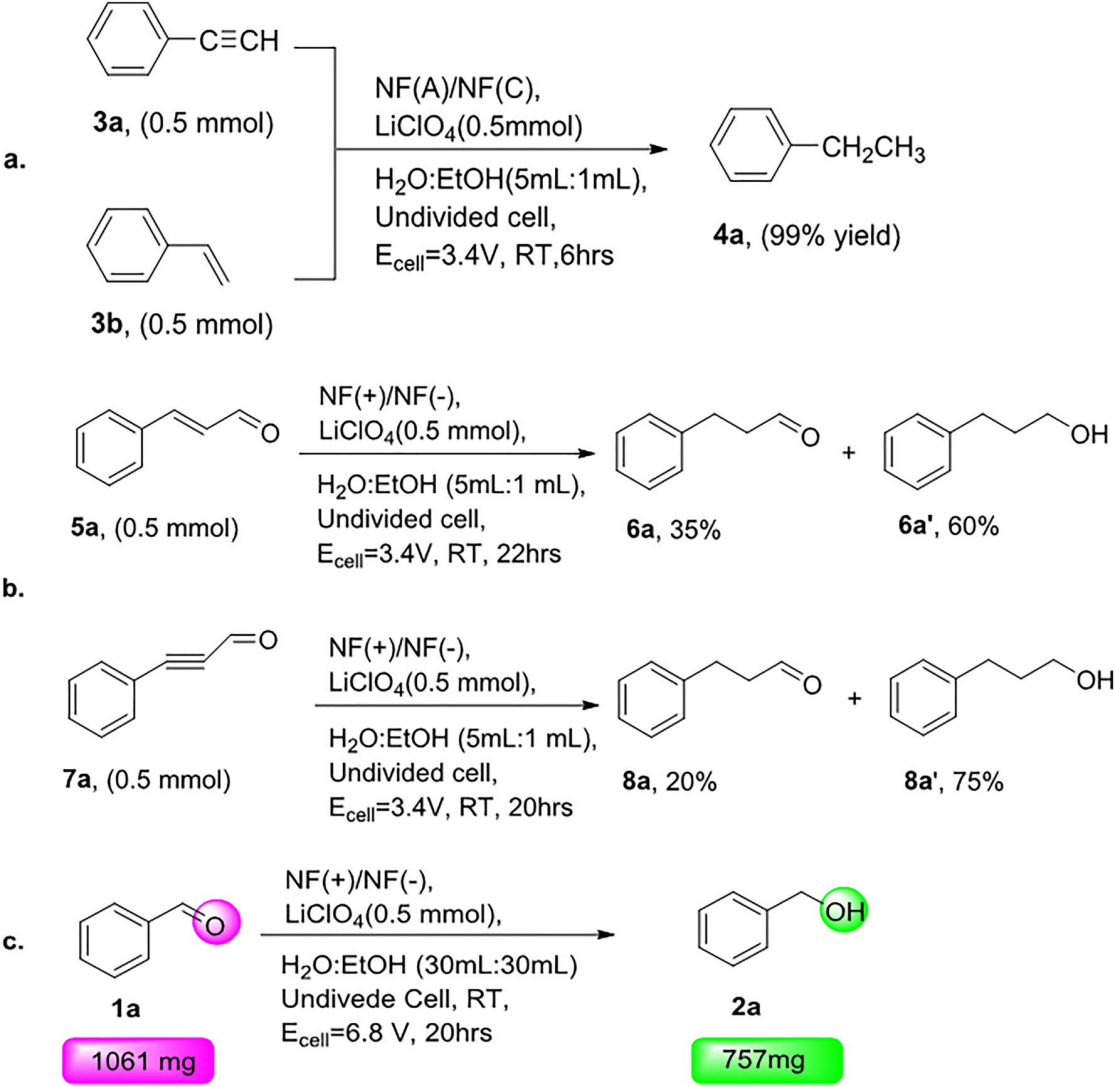
Hydrogenation of alkyne and alkene (**a**). A comparison of the activity of carbonyl and alkene/ alkyne functional group (**b**). Scaling up the hydrogenation reaction(**c**).

To find further applications for the procedure, we attempted to extend the reaction scope to challenging substrates such as C=C and C≡C bond reductions. The hydrogenation of alkene and alkyne has also been an essential tool in synthesizing medicines and pharmaceutical industries^48,49^. However, these reactions often require the use of noble transition-metal catalysts (e.g. Pd and Pt) or Renay-Ni, which is less expensive albeit hard to handle^50–52^. The summarized -electrochemical hydrogenation of unsaturated organic compounds reports were collected in a review article by Ye in 2021^53^. Under the present system, styrene and phenylacetylene afforded ethylbenzene in a 99% yield. Further investigation indicates a low level of selectivity found under our method for the C=O bond reduction over C=C hydrogenation. To verify this claim, we investigated the reaction of cinnamaldehyde and 3-phenylpropiolaldehyde. The GC results showed that hydrogenation of C=C was faster than C=O reduction (Fig. 2a,b). To demonstrate the scalability of this new technology, a 1.061 g scale reduction of benzaldehyde was performed using inexpensive nickel foam as anode and cathode in a breaker, and the corresponding product 2a resulted in a near quantitative 70% isolated yield (Fig. 2c).

**Figure 3:**
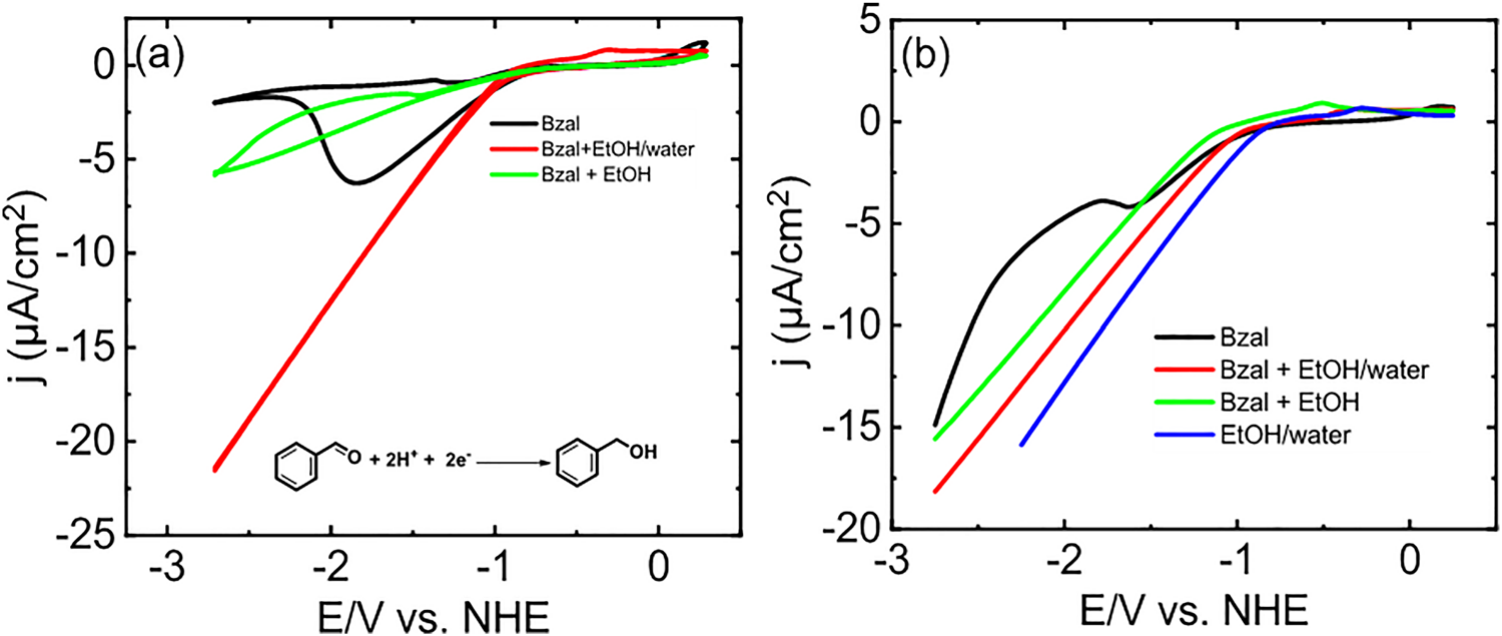
(**a**) Cyclic voltammograms of benzaldehyde (black), benzaldehyde in EtOH (green), and benzaldehyde in EtOH: H_2_O (red) on nickel foam. (**b**) Linear sweep voltammetry scans (LSV) with LiClO_4_ and NH_4_PF_6_ as the supporting electrolyte when containing benzaldehyde (black), benzaldehyde in EtOH (green), EtOH: H_2_O (blue), and benzaldehyde in EtOH and H_2_O (red). Scan rate set at 20 mV/s with a nickel foam working electrode. An Ag/AgCl electrode was used as the reference and a nickel foam as the counter electrode.

We observed a featureless cyclic voltammogram without any cathodic peak for a mixture of EtOH/ benzaldehyde and EtOH/ H_2_O/ benzaldehyde, whereas cyclic voltammetry recorded from benzaldehyde displayed a new cathodic peak near − 2.2 V (Fig. 3a). Likewise, Fig. 3b exhibits linear sweep voltammograms (LSV) prepared using electrolyte solutions containing benzaldehyde, EtOH, EtOH/ H_2_O, and a mixture of EtOH/ H_2_O/ benzaldehyde, suggesting that benzaldehyde reduction takes place at near − 2.0 V (see more details in supporting information)^18^.

Although the mechanism of carbonyl reduction was not discussed herein, the experimental and theoretical study of benzaldehyde reduction by Rousseau and Lilga’s group strongly implied that in protic solvents (H_2_O or EtOH), alcohol is the favorite product.

**Figure 4:**
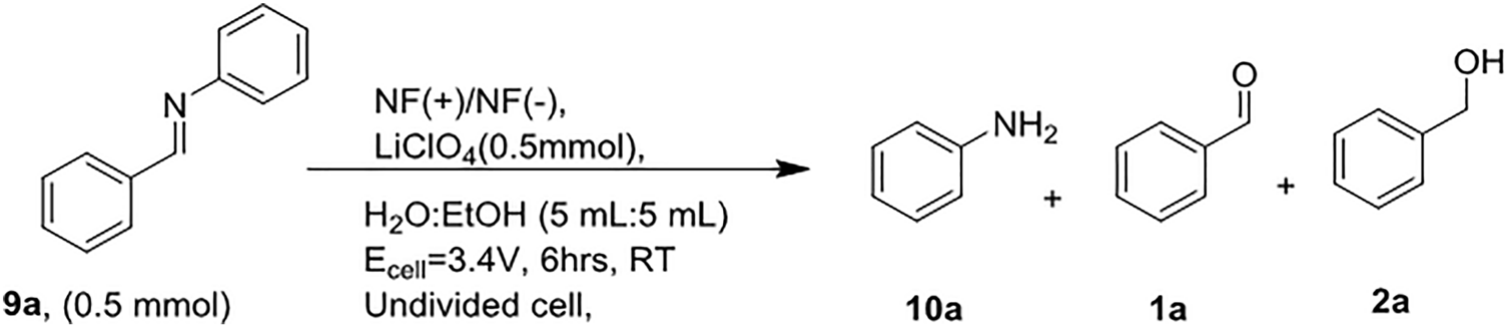
Electrochemical reduction of N-benzyl-1-phenylmethanimine.

Unlike protic solvents in aprotic conditions, dimerization was preferred, and the pinacol product was obtained^18^. In addition, the current electrochemical methodology failed to produce stable C–N products for the reduction of imines such as *N*-benzylideneaniline. However, aldehyde reduction afforded only trace quantities of benzyl alcohol (Fig. 4).

A comparison of direct cathodic reduction of benzaldehyde with other chemical and electrochemical methods is summarized in Table 3.

**Table 3:** Comparison of the efficiency of the present method with other previous methods for reducing benzaldehyde.


Conditions	Yield (%)
PhCHO (0.5 mmol/dm^3^), CH_3_OH/CH_3_O^−^Na^+^ (1.0 mol/dm^3^), Pt (C)/Pt (A), j = 50 mA/cm^2^^56^	96
PhCHO (0.1 mol), H_2_O/MeOH (1:1), NH_4_Cl (0.2 M), j = 175 to 350 mA/ dm^2^, Ni sacrificial (A)/Ni (C)^57^	99
PhCHO (0.1 mol), H_2_O (6.0 mL), NH_4_Cl (1.0 mol/dm), j = 280 mA/dm^2^, Ni sacrificial (A)/Ni (C), ultrasound	99
*B*-CyD solution [(4.0 mL of 7.0 mmol/dm^3^)^55^	
PhCHO (1.0 mmol), H_2_O/MeOH (10 mL), NH_4_Cl (0.2 mmol), Ni(A)/Fe, Ni or Cu(C), Charge (2 F /mol)^54^	90–95
PhCHO (0.25 mmol), sp^3^‐sp^3^ Diboron (0.5 mmol), H_2_O (1.0 mL), 20 °C, 18 hrs^58^	99
PhCHO (2.0 mmol), (EtO)_3_SiH (2.2 mmol), [2,6- (iPr_2_PO)_2_C_6_H_3_]Fe(H)(PMe_3_)_2_ (0.020 mmol), THF (2.0 mL), 50 °C, 1.5 h then NaOH (10%), MeOH, 50 °C^59^	91
PhCHO, TBAPF_6_ (0.1 M), 1,2-difluorobenzene, E_app_ = − o.51 V, (C_5_Ph_4_OH)Mo(CO)_3_(CH_3_CN)^+^[OTf]^60^	67
PhCHO (1.0 mmol),LiAlH_4_ (0.5 mmol), Silica Chloride (0.5 mmol), dry diethyl ether (10 ml), RT, 10 min^61^	100
PhCHO (0.5 mmol), NF (C)/NF (A), LiClO_4_ (0.5 mmol), H_2_O/EtOH (6.0 mL), Undivided Cell, RT, 6 h, E_cell_ = 3.4 V (This work)	100

## Conclusions

In summary, an innovative electrochemical protocol for reducing carbonyl groups to the corresponding alcohols was developed. The reaction was performed under external reductant–free conditions to provide a broad range of alcohol and alkane compounds in good yields. Furthermore, the scalability of our procedure was confirmed with the reduction of benzaldehyde on a 1.0 g scale. We believe this green and mild approach to preparing alcohols and alkanes will apply in academic and industrial modern fields.

## Methods

### Materials

All chemical materials were used without further purification. Lithium perchlorate (LiClO_4_), benzaldehyde, 4-methoxybenzaldehyde, 4-isopropyl benzaldehyde, 4-chlorobenzaldehyde, 3-chlorobenzaldehyde, 2,4-dichlorobenzaldehyde, 2,6-dichlorobenzaldehyde, 2, 4-dimethylbenzaldehyde, 2, 4-dimethoxybenzaldehyde, cyclohexanone, hexanal, 2-chlorobenzaldehyde, 3-chlorobenzaldehyde, 4-cyanobenzaldehyde, 4-methylbenzaldehyde, 1-naphthaldehyde, 2-naphthaldehyde, acetophenone, benzophenone, cinnamaldehyde, and solvents were purchased from Sigma-Aldrich or Merck Company. 3-phenylpropiolaldehyde was synthesized according to the procedure followed by Kim^47^. The products were characterized by gas chromatography (GC) and thin-layer chromatography (TLC). Preparative thin layer chromatography (PTLC) separations were carried out on 0.25 or 0.5 mm E. Merck silica gel plates (60F-254).

The nickel foam (Nanobazar), iron foam (Suzhou JSD foam metal Co., Ltd), iron-nickel foam (Suzhou JSD foam metal Co., Ltd), aluminum foil (Suzhou JSD foam metal Co., Ltd), and titanium foil (Suzhou JSD foam metal Co., Ltd) purchased from commercial sources. The electrochemical reactions were conducted through a Model JPS-302D tracking dual DC power supply. Cyclic voltammetry analysis was carried out on a Palmsens (Emstat3 +) electrochemical workstation, using a nickel foam as the working electrode, a Pt electrode as a counter electrode, and an Ag/AgCl as a reference electrode. The cyclic voltammogram was recorded at a scan rate of 20 mV/s. GC yield was performed on a Model TOF LC/MS gas chromatograph spectrometer. ^1^H NMR spectra were measured in CDCl_3_ using a 400 MHz spectrometer (Bruker, Switzerland). ^13^C NMR spectra were measured in CDCl_3_ at 101 MHz. The following abbreviations were used to explain the multiplicities: s = singlet, d = doublet, t = triplet, q = quartet, m = multiple.

### General experimental procedure

In a 10 ml beaker with a stirring bar, the starting material (0.5 mmol), LiClO_4_ (0.5 mmol) in 6.0 mL H_2_O, and EtOH (5:1) were added successively. The electrolytic cell was equipped with nickel foam (1 cm × 1 cm) as an anode and a cathode. The solution was electrolyzed at ambient temperature under a constant voltage (3.4 V). After completing the reaction, the mixture was extracted with ethyl acetate. The obtained organic layer dried over Na_2_SO_4_ and was used for GC and NMR spectroscopy.

### Procedure for gram-scale synthesis of 2a

In a 100 ml beaker with a stirring bar, the starting material (10.0 mmol), LiClO_4_ (5.0 mmol) in 60 mL H_2_O, and EtOH (5:1) were added successively. The electrolytic cell was equipped with nickel foam (5 cm × 5 cm) as an anode and a cathode. The solution was electrolyzed at ambient temperature under a constant voltage (6.8 V). After completing the reaction, the mixture was extracted with ethyl acetate. The obtained organic layer dried over Na_2_SO_4_ and was used for GC and NMR spectroscopy.

## Supplementary Information

Supplementary Information.

## Data Availability

All data generated or analyzed during this study are included in this published article [and its Supplementary Information files].
