# Supplementary material for: Water oxidation couples to electrocatalytic hydrogenation of carbonyl compounds and unsaturated carbon–carbon bonds by nickel
Source: Sci Rep. 2022 Nov 19;12:19968. doi: 10.1038/s41598-022-23777-7 (PMC9675855; doi:10.1038/s41598-022-23777-7)
Supplement: Supplementary file 1 — Supplementary Information. [file 41598_2022_23777_MOESM1_ESM.docx]

**Supporting Information**

**Water Oxidation Couples to Electrocatalytic Hydrogenation of Carbonyl Compounds and Unsaturated Carbon-Carbon bonds** **by Nickel**

Leila Behrouzi^a^, Zahra Zand^a^, Mobina Fotuhi^a^, Babak Kaboudin^a^* and Mohammad Mahdi Najafpour^a -c^*

^a^ Department of Chemistry, Institute for Advanced Studies in Basic Sciences (IASBS), Zanjan, 45137-66731, Iran

^b^ Center of Climate Change and Global Warming, Institute for Advanced Studies in Basic Sciences (IASBS), Zanjan, 45137-66731, Iran

^c^ Research Center for Basic Sciences & Modern Technologies (RBST), Institute for Advanced Studies in Basic Sciences (IASBS), Zanjan, 45137-66731, Iran

* Corresponding authors: Phone: (+98) 24 3315 3220; [kaboudin@iasbs.ac.ir](mailto:kaboudin@iasbs.ac.ir); Phone: (+98) 24 3315 3201; E-mail: [mmnajafpour@iasbs.ac.ir](mailto:mmnajafpour@iasbs.ac.ir)

**1. ^13^C NMR and ^1^H NMR of products**……………………………………………………………….…. page 3

**2. ^13^C NMR and ^1^H NMR spectra of products**

^13^C NMR and ^1^H NMR spectrum of Benzyl alcohol………………………………………………………page 4

^13^C NMR and ^1^H NMR spectrum of 3-Cholorobenzyl alcohol…………………………………………… page 5

^13^C NMR and ^1^H NMR spectrum of 4-Methoxybenzyl alcohol…………………………………………...page 6

^13^C NMR and ^1^H NMR spectrum of 2, 4-Dichlorobenzyl alcohol ………………………………………..page 7

^13^C NMR and ^1^H NMR spectrum of 2-Naphthylmethanol ………………………………………………. page 8

^13^C NMR and ^1^H NMR spectrum of Phenylethanol ………………………………………………………page 9

**3. GC Analysis Results:**

GC analysis of Benzaldehyde reduction……………………………………………...…......................... page 10

GC analysis of 2-Bromobenzaldehyde reduction… ………………………………...…......................... page 11

GC analysis of 4-Chlorobenzaldehyde reduction………………… ………………...…......................... page 12

GC analysis of 4-Isopropylbenzaldehyde reduction……………………………………… ..................... page 13

GC analysis of 4-Methoxybenzaldehyde reduction………… ………………………………................ page 14

GC analysis of 2,4-Dichlorobenzaldehyde reduction……………………………….…........................... page 15

GC analysis of 2,6-Dichlorobenzaldehyde reduction………………… …………...…........................... page 16

GC analysis of 2,4-Dimethylbenzaldehyde reduction……………………… ……...…........................... page 17

GC analysis of 1-Naphthaldehyde reduction………………………………………...….......................... page 18

GC analysis of 2-Naphthaldehyde reduction………………………………………...….......................... page 19

GC analysis of Acetophenone reduction……………………..……………………...….......................... page 20

GC analysis of Benzophenone reduction…………………………………………...…........................... page 21

GC analysis of Cyclohexanone reduction…………………………………………...….......................... page 22

GC analysis of 1-Hexanal reduction……………………………………………...….............. .............. page 23

GC analysis of Styrene reduction……………….…………………………………...….......................... page 24

GC analysis of Phenylacetylene reduction…………………………………………...….......................... page 25

**4. Cyclic Voltammetry and Linear Sweep Voltammetry results**……………….… ...................... page 26,27

Reference…………………………………………………………………………………………..…….. page 28

**1. ^13^C NMR and ^1^H NMR of the products:**

| **Benzyl alcohol** | The characterization data obtained for benzyl alcohol were identical to those previously reported in the literature^[^[^1^](#_ENREF_1)^]^. ^1^H NMR (400 MHz, CDCl_3_): δ 7.40- 7.28 (m, 5H), 4.73(d, 2H), 1.75 (t, 1H).^13^C NMR (101 MHz, CDCl_3_): δ 140.96, 128.55, 127.62, 126.86, 65.09. |
| --- | --- |
|  |  |
| **3-Cholorobenzyl alcohol** | The characterization data obtained for 3-cholorobenzyl alcohol were identical to those previously reported in the literature^[^[^2^](#_ENREF_1)^]^. ^1^H NMR (400 MHz, CDCl_3_): δ 7.40 (s, 1H), 7.34- 7.25 (m, 3H), 4.71 (s, 2H), 1.74 (s, 1H). ^13^C NMR (101 MHz, CDCl_3_): δ 142.86, 134.32, 129.65, 127.51, 126.92, 124.78, 64.27. |
|  |  |
| **4-Methoxybenzyl alcohol** | The characterization data obtained for 4-methoxybenzyl alcohol were identical to those previously reported in the literature^[^[^1^](#_ENREF_1)^]^. ^1^H NMR (400 MHz, CDCl_3_): δ 7.26 (d, 2H), 6.89 (d, 2H), 4.53 (s, 2H), 3.79 (s, 3H), 3.15 (s, 1H). ^13^C NMR (101 MHz, CDCl_3_): δ 159.13, 132.44, 128.47, 113.73, 64.58, 55.11. |
|  |  |
| **2, 4-Dichlorobenzyl alcohol** | The characterization data obtained for 2,4-dicholorobenzyl alcohol were identical to those previously reported in the literature^[^[^4^](#_ENREF_1)^]^. ^1^H NMR (400 MHz, CDCl_3_): δ 7.46- 7.28 (m, 3H), 4.77 (d, 2H), 2.08 (t, 1H). ^13^C NMR (101 MHz, CDCl_3_): δ 136.75, 133.83, 133.18, 129.42, 129.13, 127.29, 62.03. |
|  |  |
| **2-Naphthylmethanol** | The characterization data obtained for 2-naphthylmethanol were identical to those previously reported in the literature^[^[^3^](#_ENREF_1)^]^. ^1^H NMR (400 MHz, CDCl_3_): δ 7.89- 7.85 (m, 3H), 7.52- 7.50 (m, 3H), 7.28 (s, 1H), 4.91(d, 2H), 1.76 (t, 1H). ^13^C NMR (101 MHz, CDCl_3_): δ 138.29, 133.36, 132.96, 128.37, 127.89, 127.72, 126.21, 125.93, 125.46, 125.17, 65.54. |
|  |  |
| **Phenylethanol** | The characterization data obtained for phenylethanol were identical to those previously reported in the literature^[^[^3^](#_ENREF_1)^]^. ^1^H NMR (400 MHz, CDCl_3_): δ 7.41-7.25 (m, 5H), 4.94 (q, 1H), 2.10 (s, 1H), 1.53 (d, 3H). ^13^C NMR (101 MHz, CDCl_3_): δ 145.61, 128.51, 127.22, 125.68, 70.41, 25.04. |
|  |  |

**2. ^13^C NMR and ^1^H NMR spectra of products.**

**^13^C NMR spectrum of Benzyl alcohol:**

**^1^H NMR spectrum of Benzyl alcohol:**


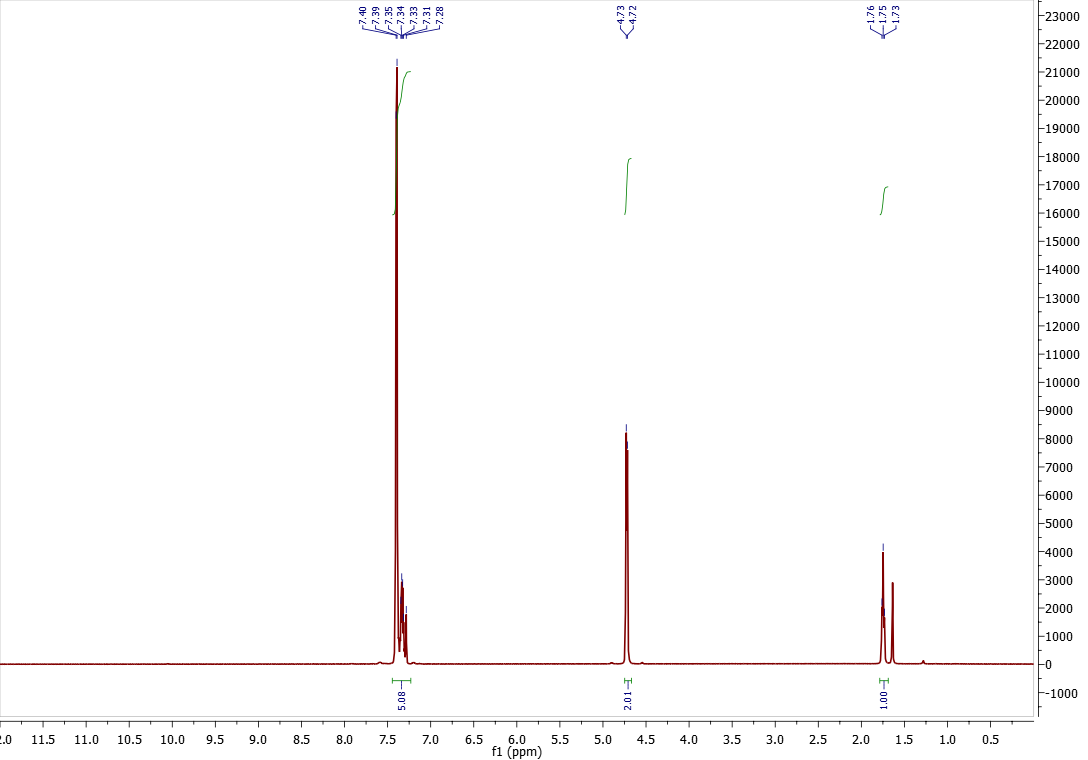

**^13^C NMR spectrum of 3-Cholorobenzyl alcohol:**


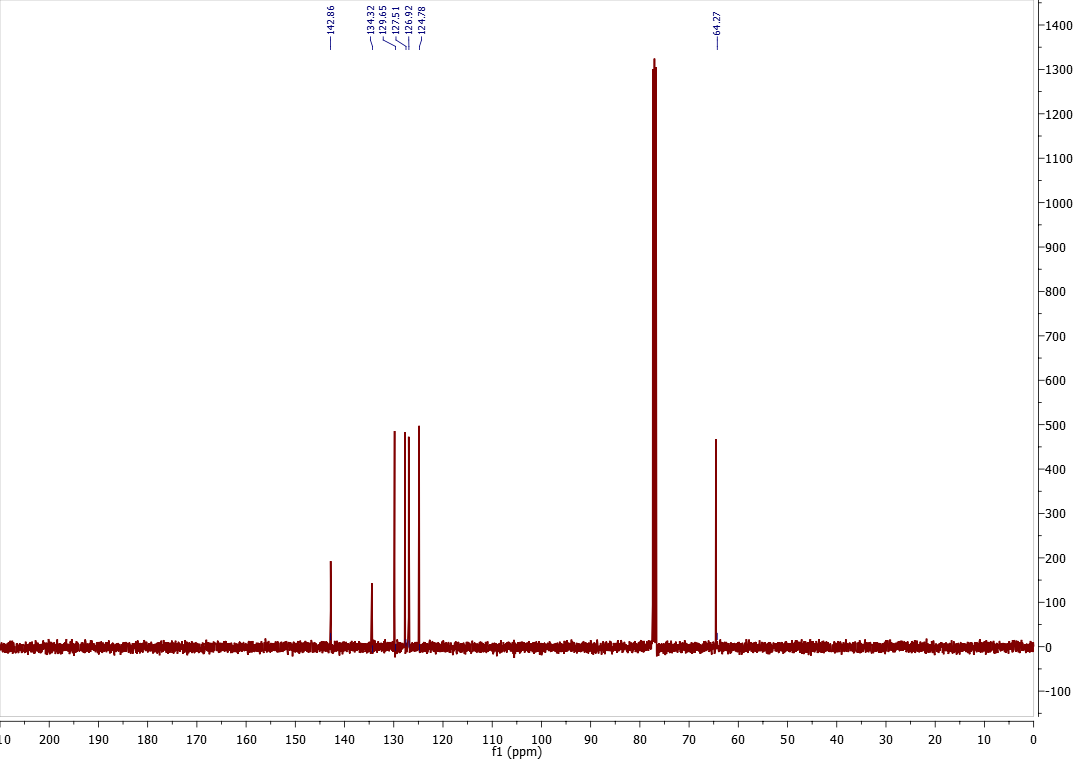

**^1^H NMR spectrum of 3-Cholorobenzyl alcohol:**


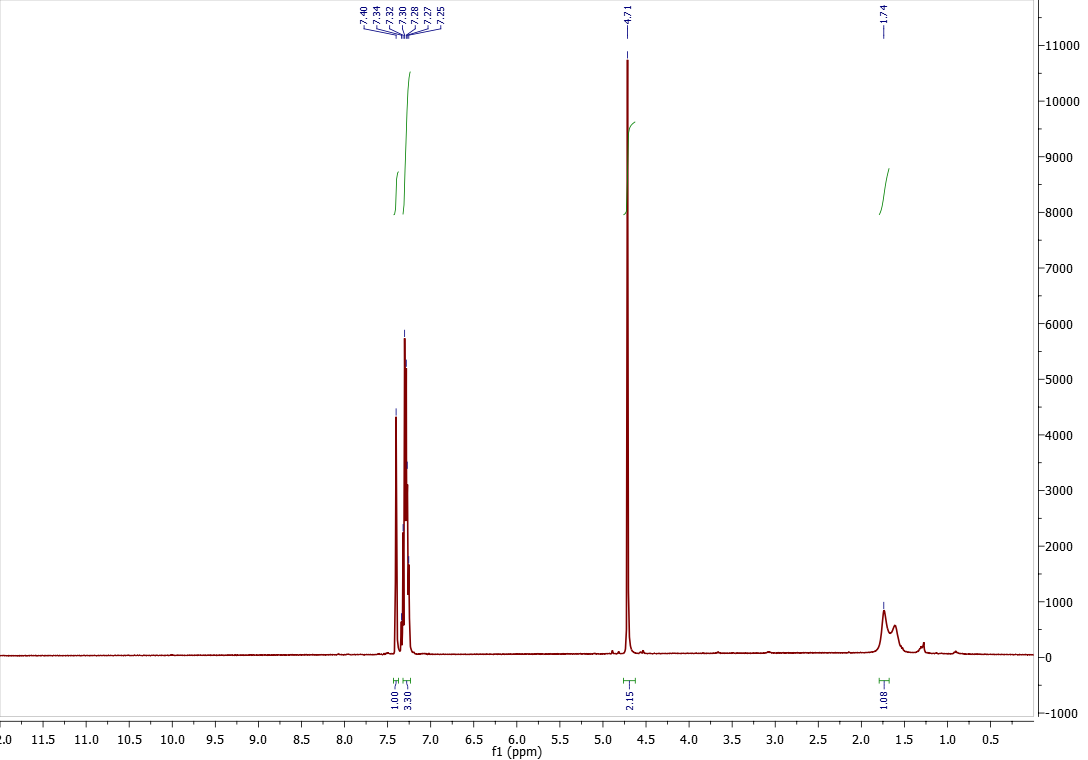

**^13^C NMR spectrum of 4-Methoxybenzyl alcohol:**


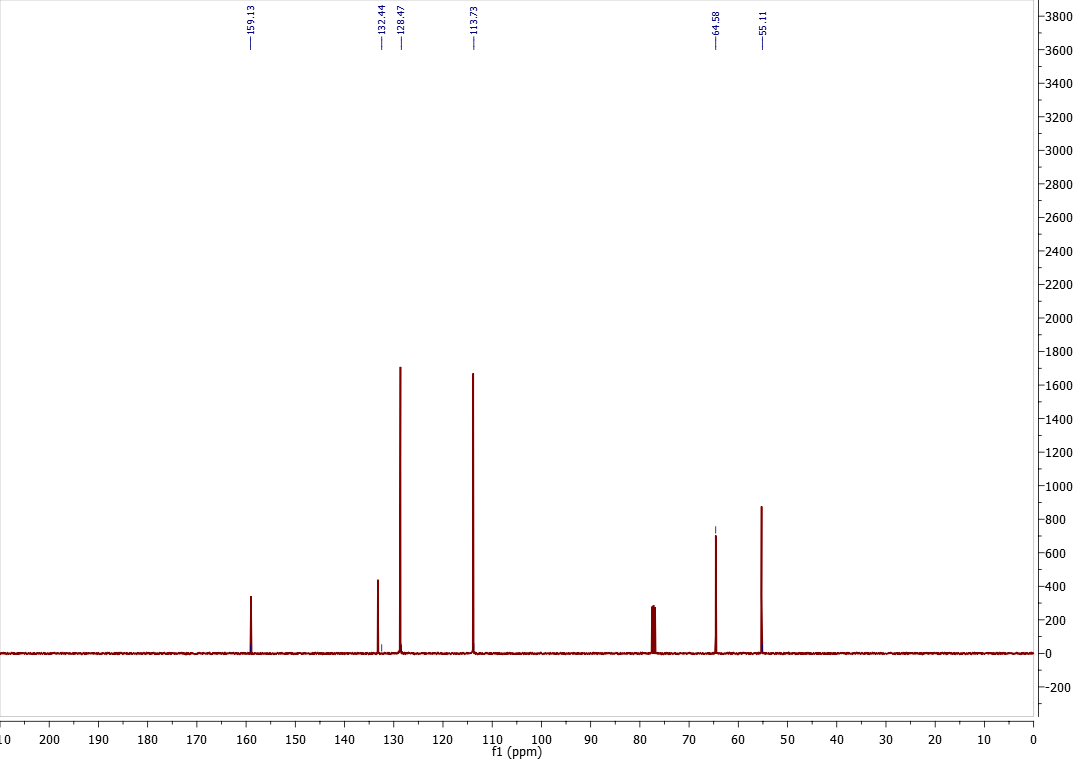

**^1^H NMR spectrum of 4-Methoxybenzyl alcohol:**


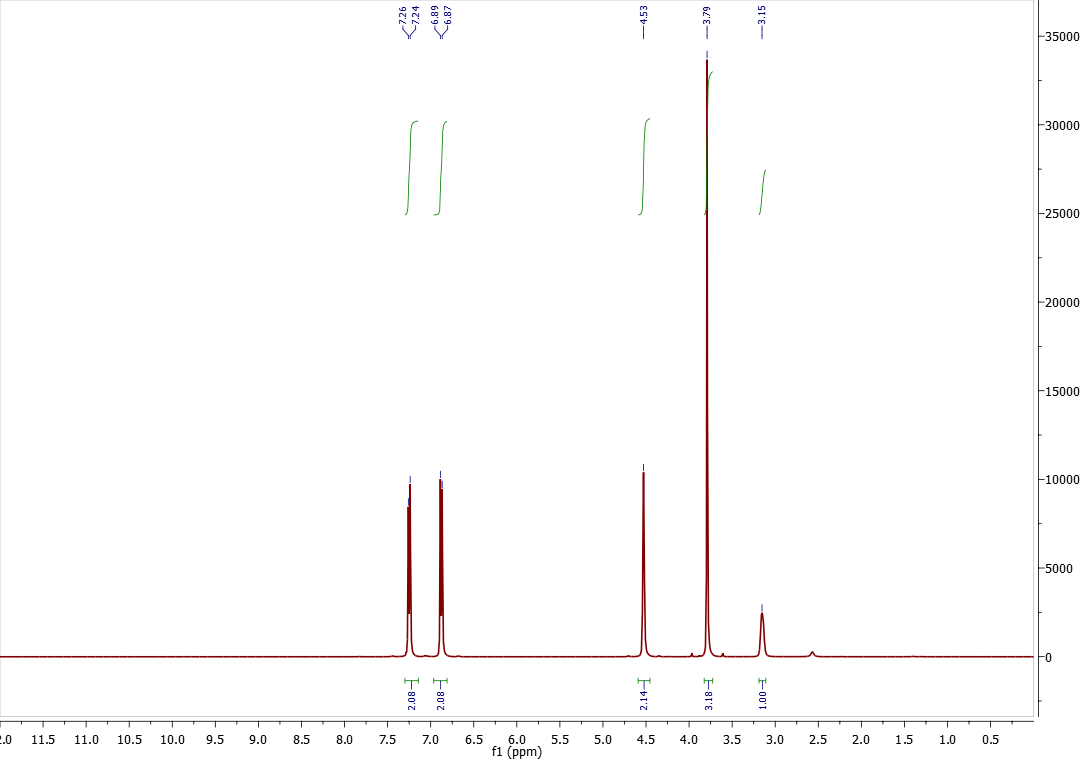

**^13^C NMR spectrum of 2, 4-Dichlorobenzyl alcohol:**


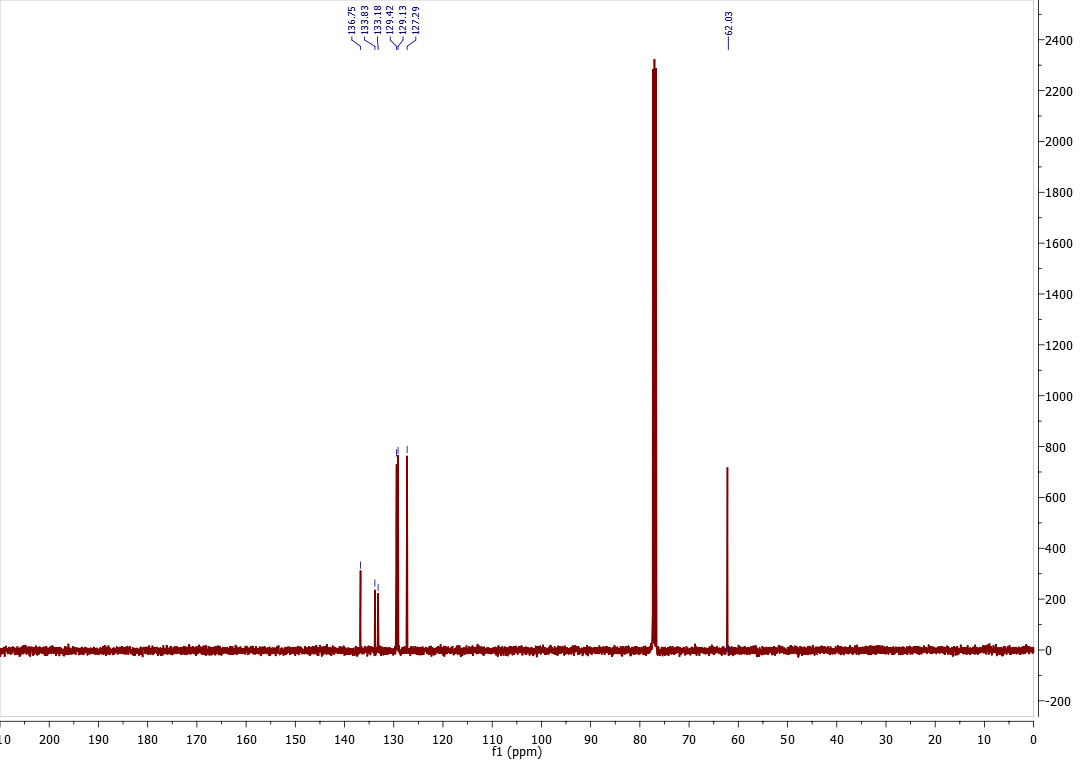

**^1^H NMR spectrum of 2, 4-Dichlorobenzyl alcohol:**


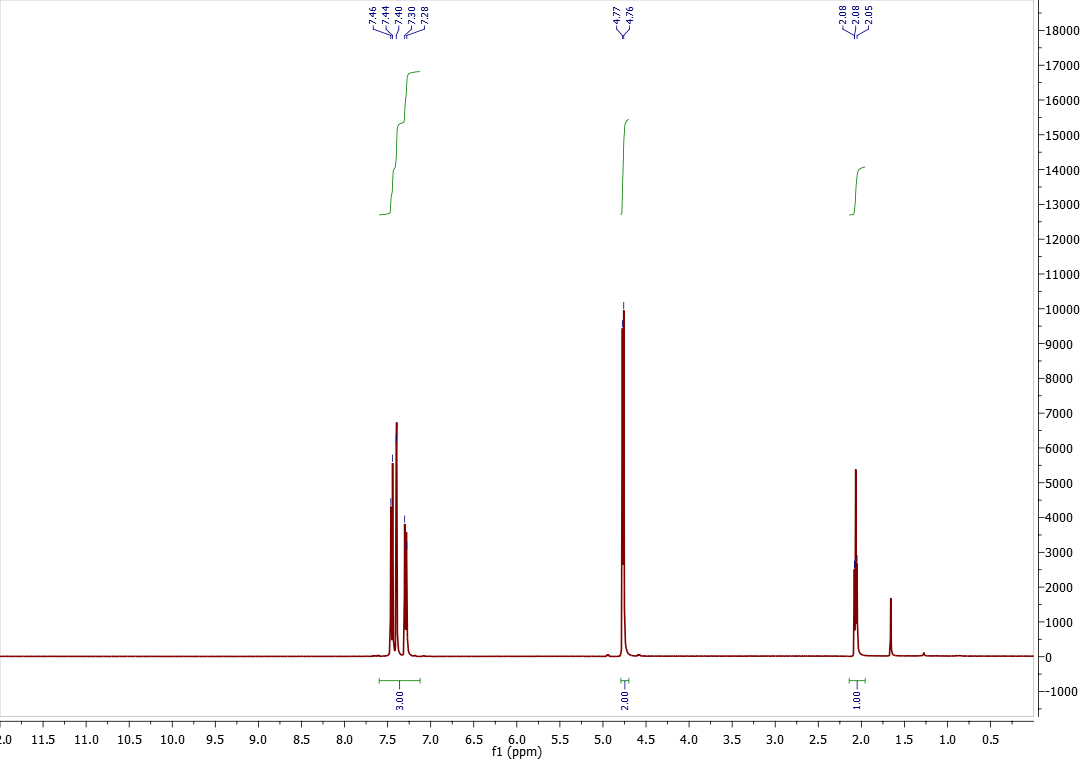

**^13^C NMR spectrum of 2-Naphthylmethanol:**

**^1^H NMR spectrum of 2-Naphthylmethanol:**


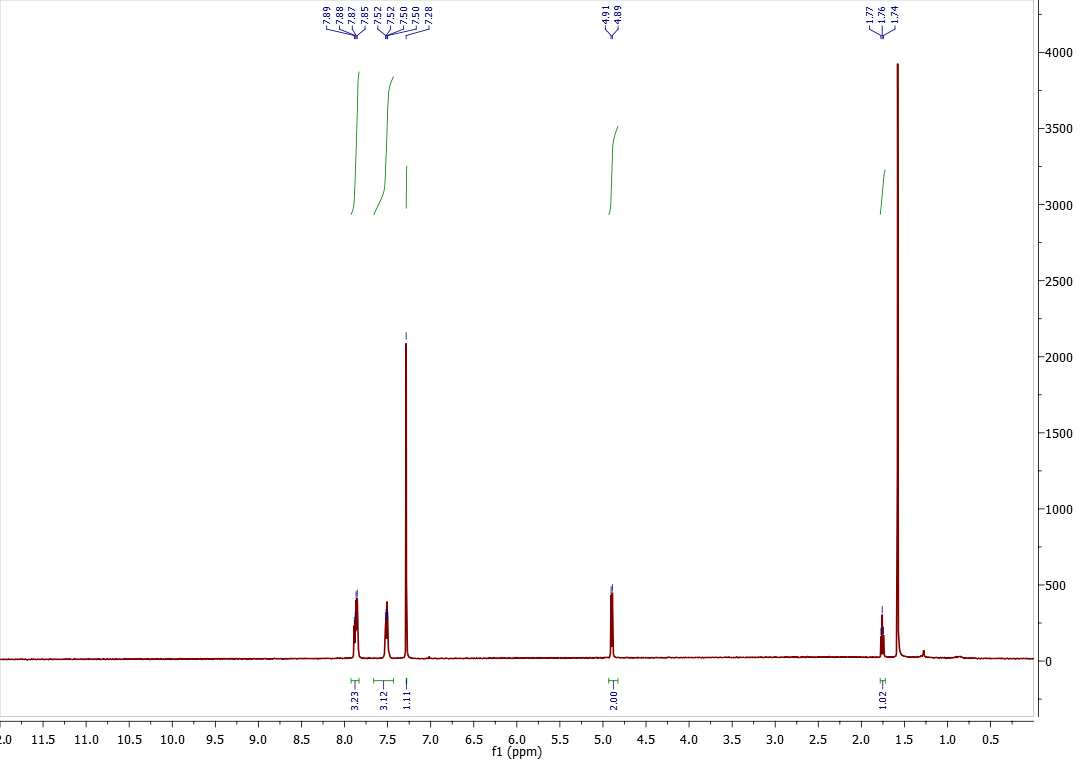

**^13^C NMR spectrum of Phenylethanol:**


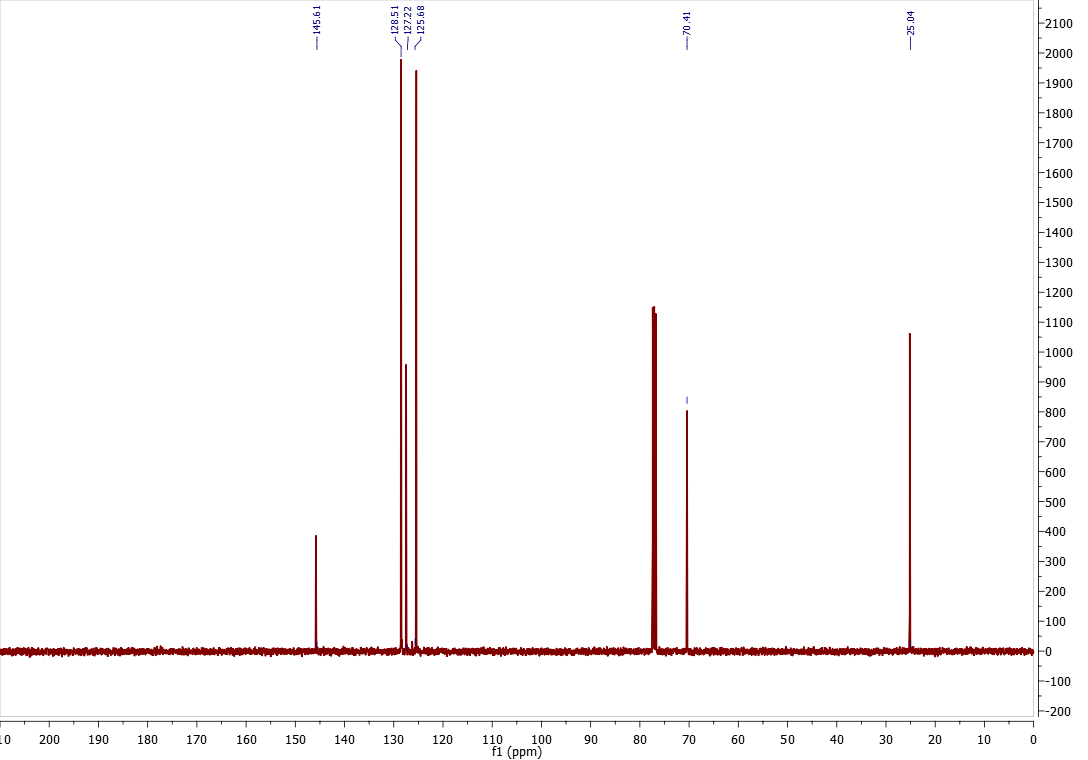

**^1^H NMR spectrum of Phenylethanol:**


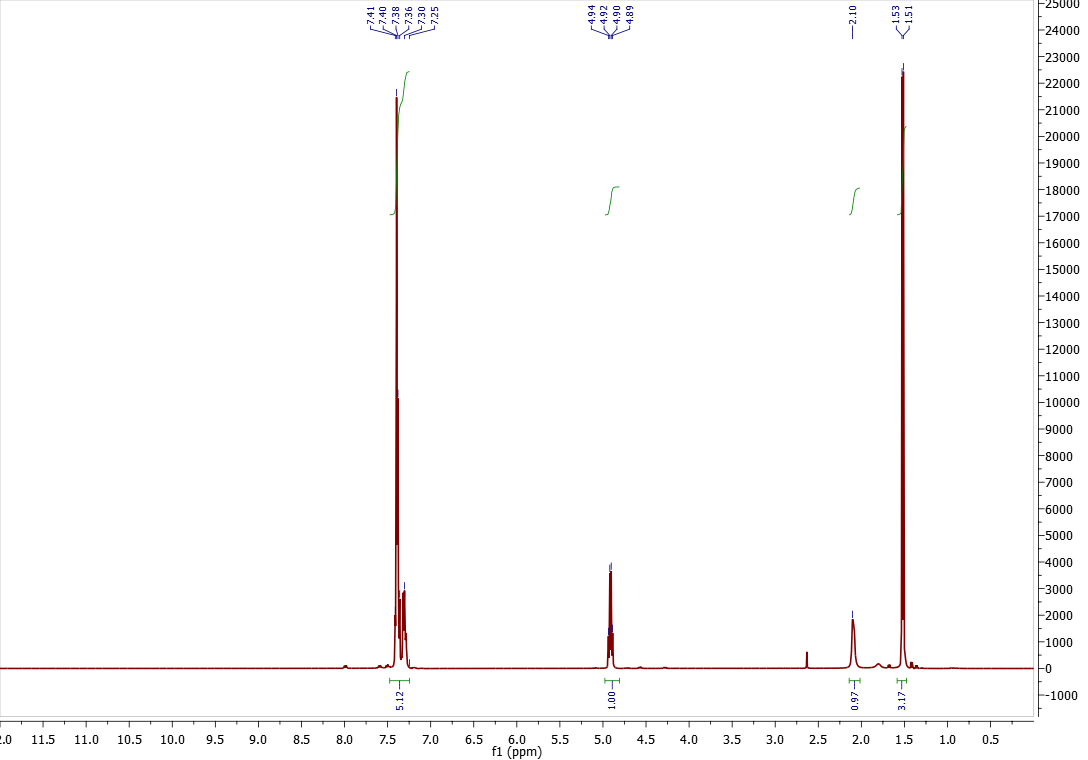

**3. GC Analysis Results:**

**GC analysis of Benzaldehyde reduction:**


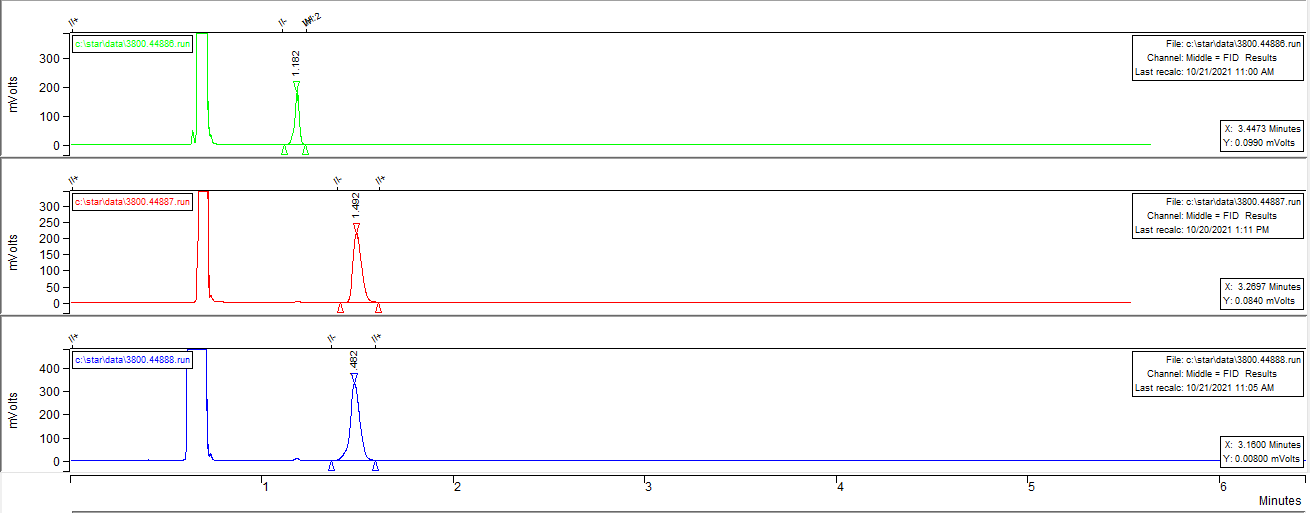


Mix of reaction

Blank of **benzyl alcohol**

Blank of **benzaldehyde**

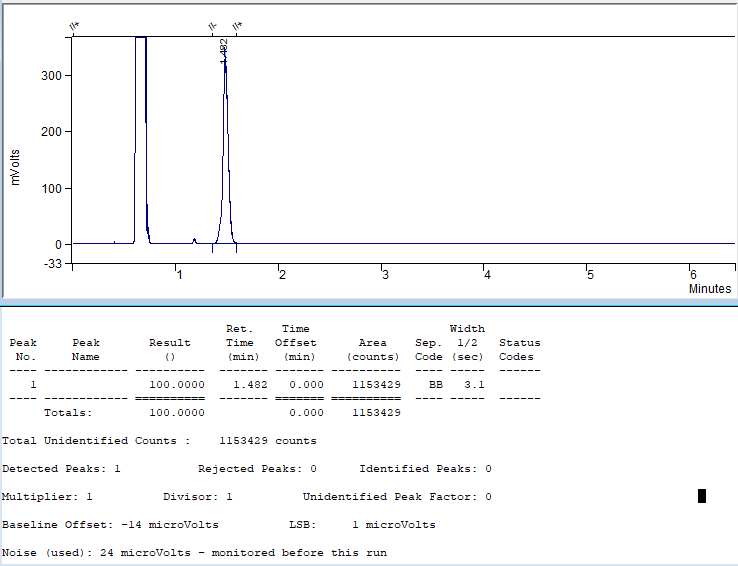

**GC analysis of 2-Bromobenzaldehyde reduction:**


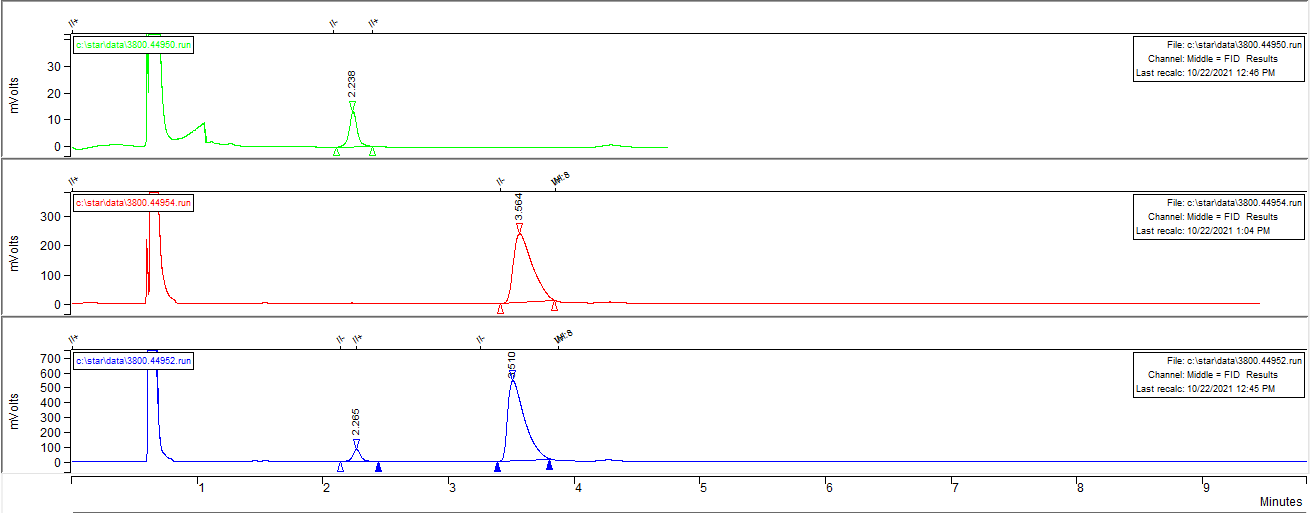


Mix of reaction

Blank of **2-bromobenzyl alcohol**

Blank of **2-bromobenzaldehyde**

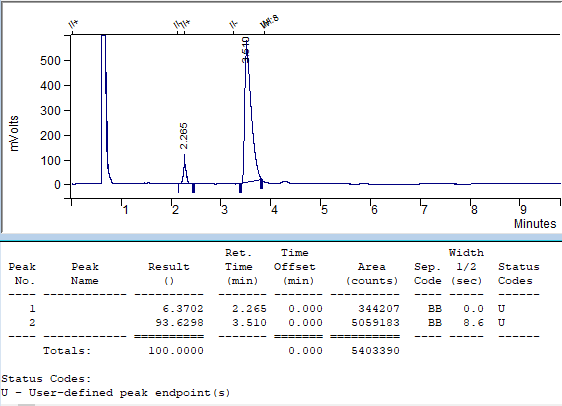

**GC analysis of 4-Chlorobenzaldehyde reduction:**


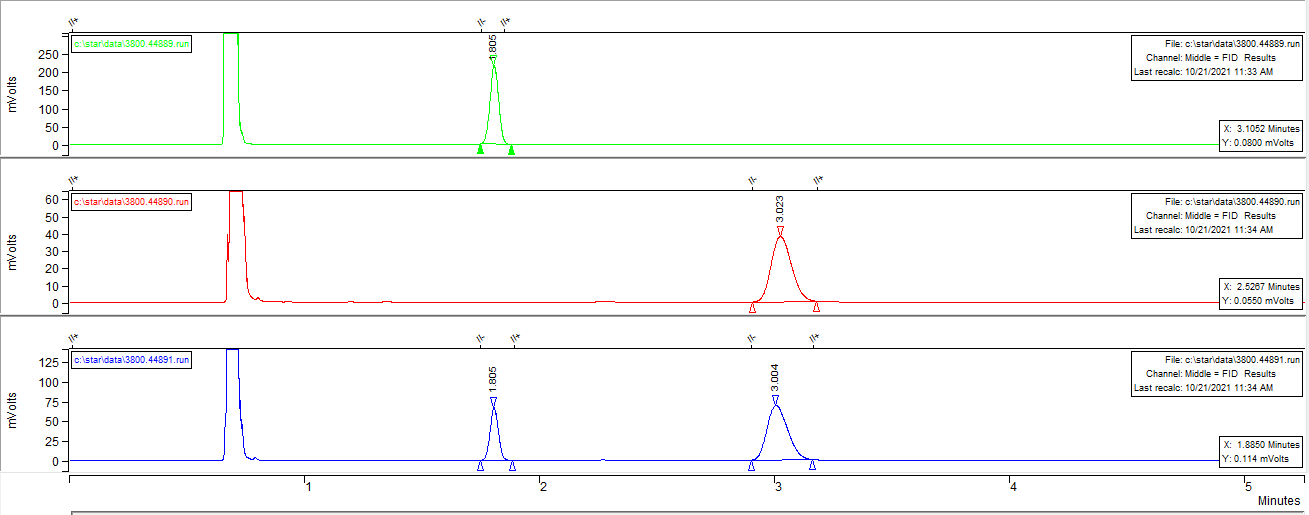

Mix of reaction

Blank of **4-chlorobenzyl alcohol**

Blank of **4-chlorobenzaldehyde**


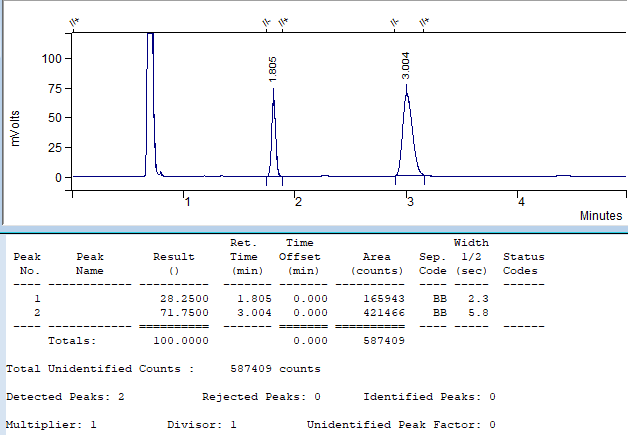

**GC analysis of 4-Isopropylbenzaldehyde reduction:**

**
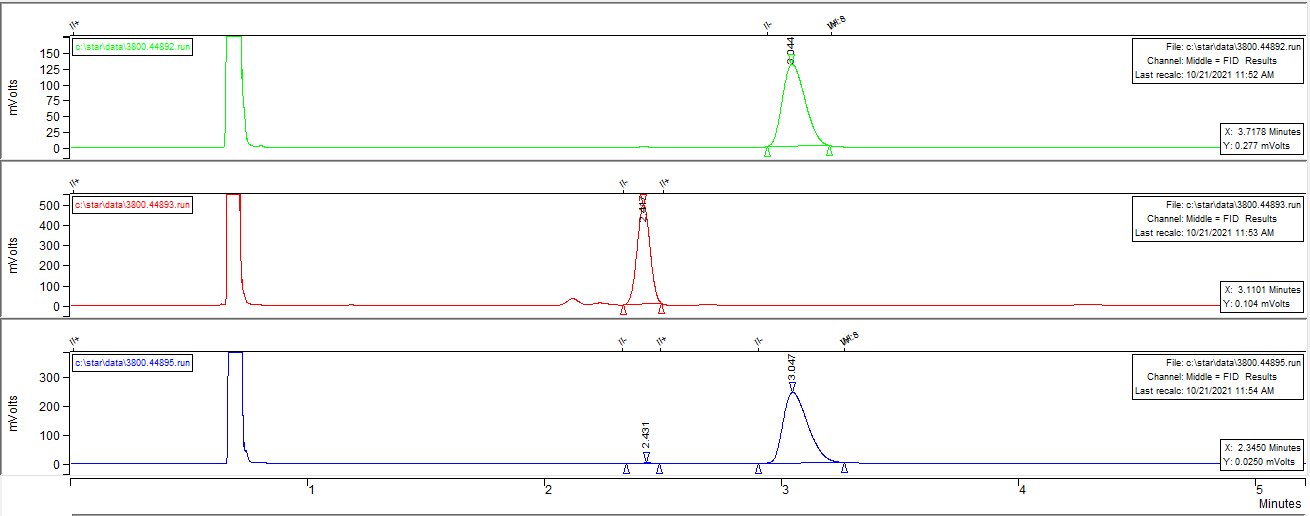
**

Mix of reaction

Blank of **4-Isopropylbenzaldehyde**

Blank of **4-Isopropylbenzyl alcohol**

**
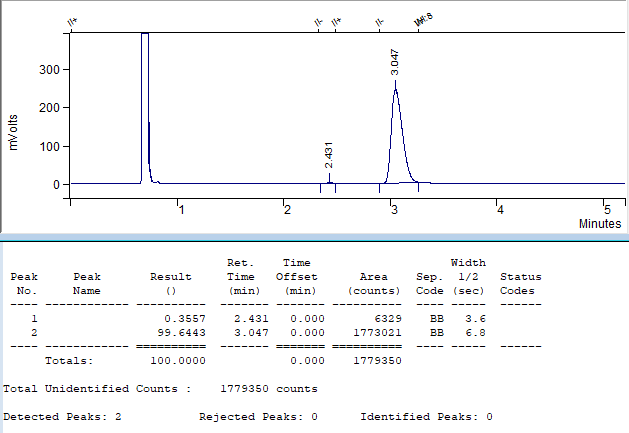
**

**GC analysis of 4-Methoxybenzaldehyde reduction:**

**
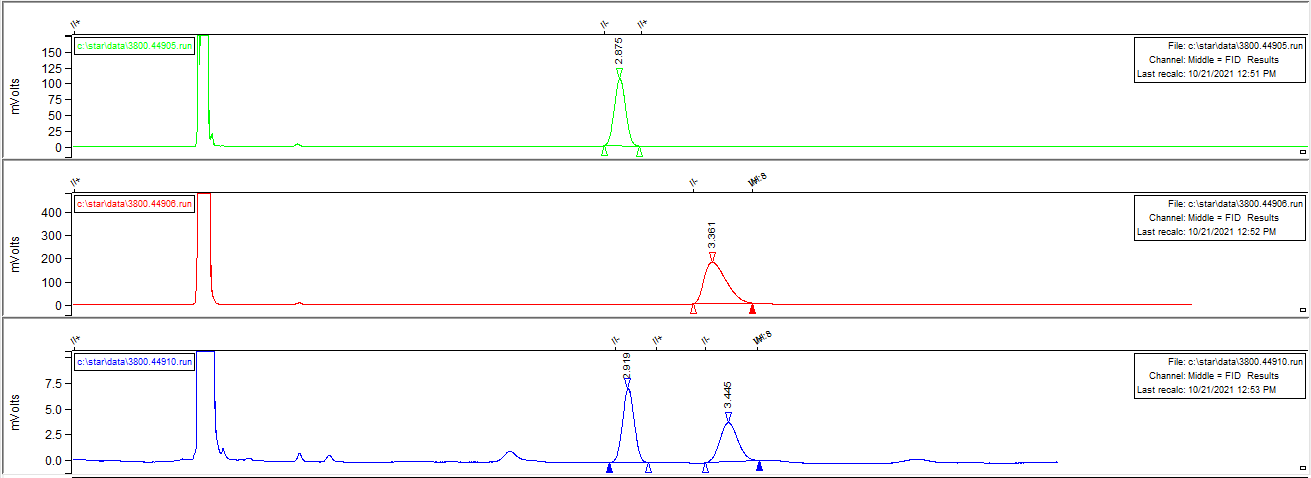
**

Mix of reaction

Blank of **4-Methoxybenzylaldehyde**

Blank of **4-Methoxybenzyl alcohol**

**
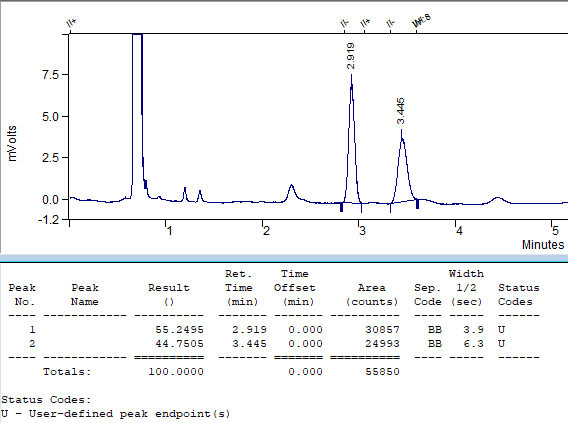
**

**GC analysis of 2,4-Dichlorobenzaldehyde reduction:**


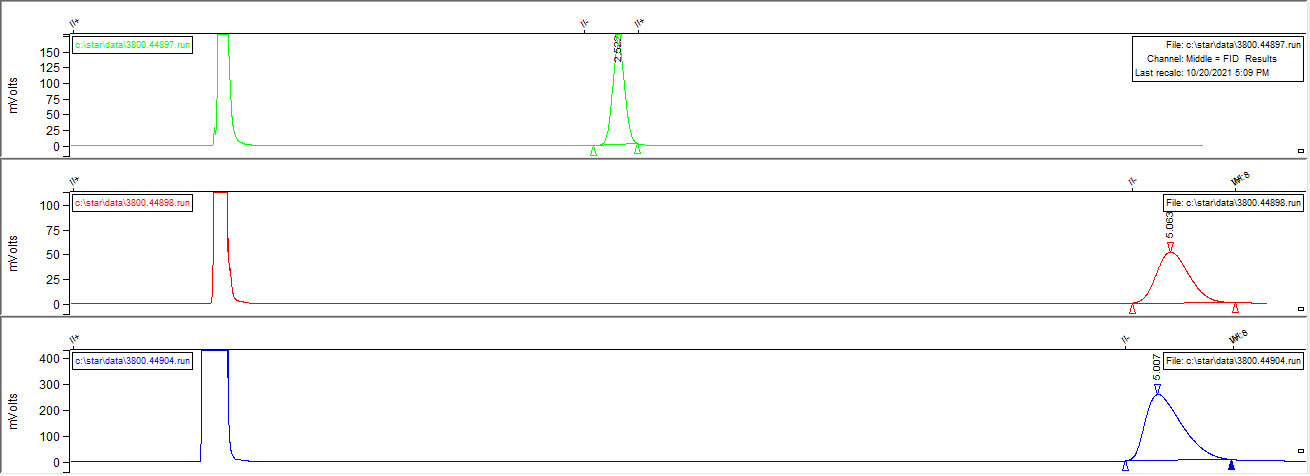


Blank of **2, 4-Dichlorobenzyl alcohol**

Blank of **2, 4-Dichlorobenzaldehyde**

Mix of reaction


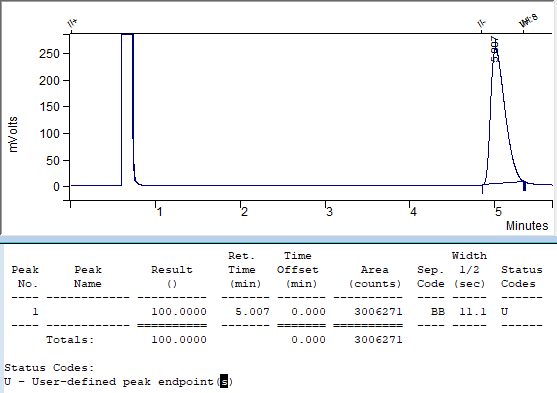

**GC analysis of 2,6-Dichlorobenzaldehyde reduction:**

**
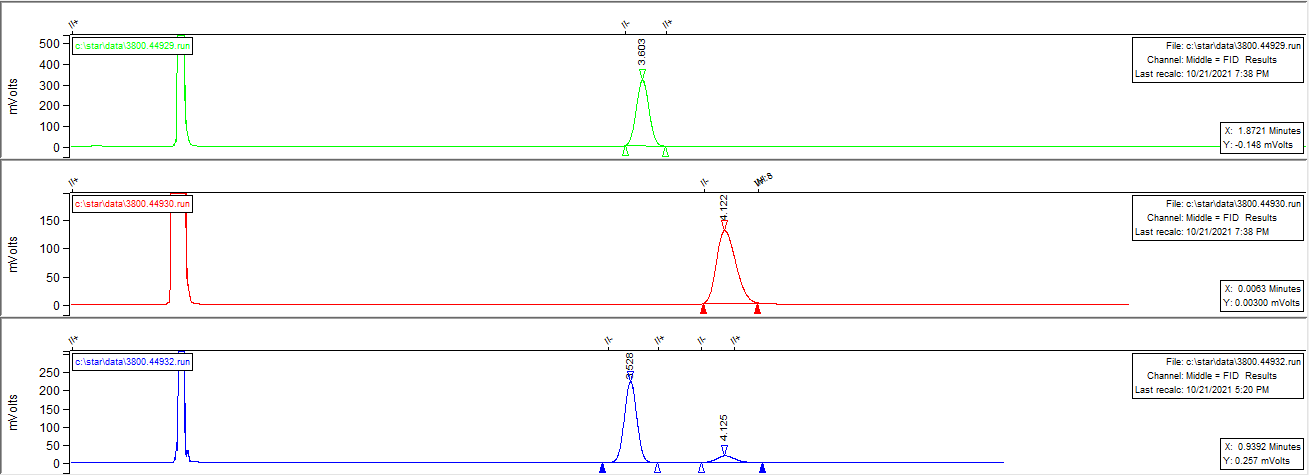
**

Mix of reaction

Blank of **2, 6-Dichlorobenzyl alcohol**

Blank of **2, 6-Dichlorobenzaldehyde**


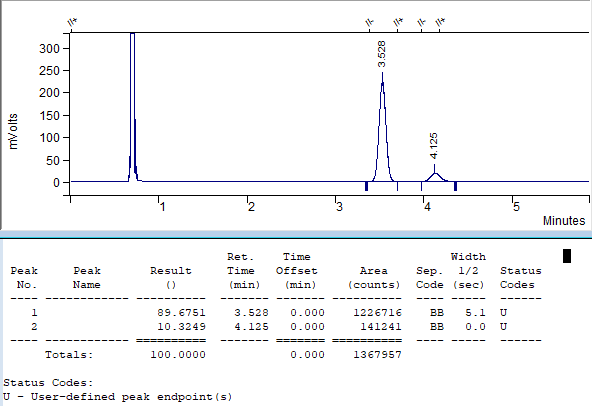

**GC analysis of 2,4-Dimethylbenzaldehyde reduction:**

**
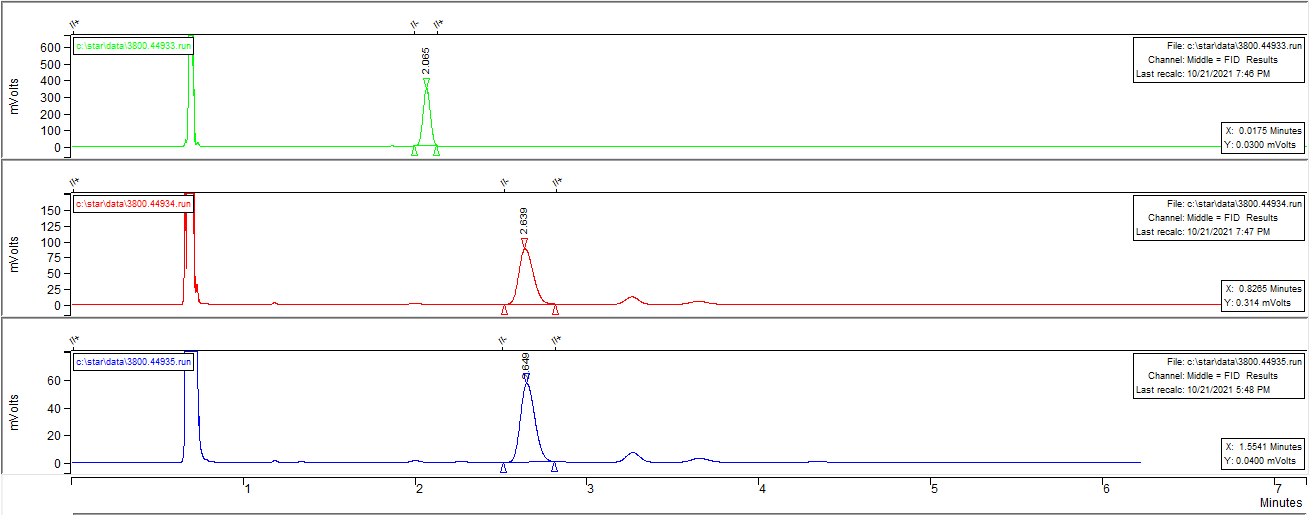
**

Blank of **2, 4- Dimethylbenzyl alcohol**

Mix of reaction

Blank of **2, 4- Dimethylbenzaldehyde**

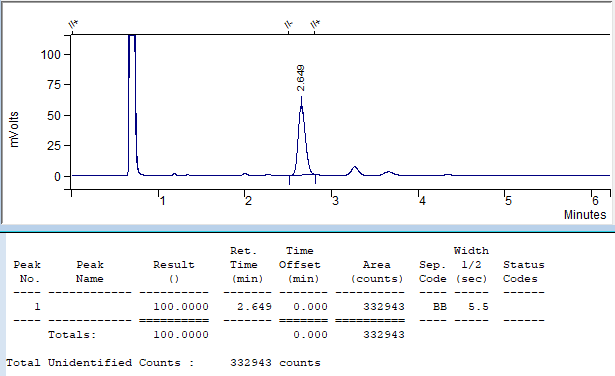

**GC analysis of 1-Naphthaldehyde reduction:**

**
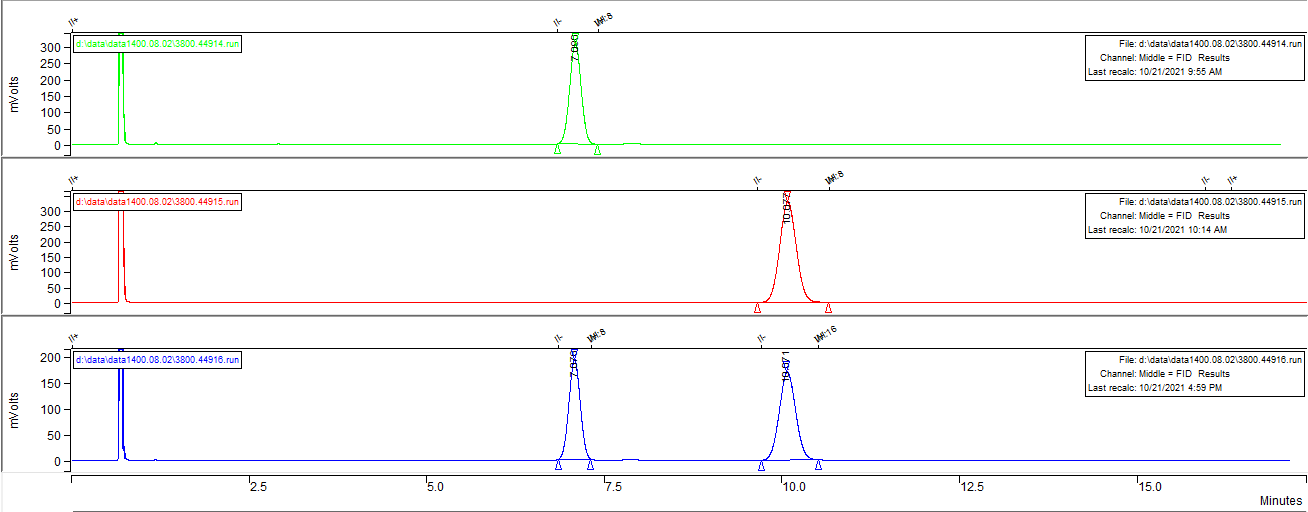
**

Blank of **1-Naphthylmethanol**

Mix of reaction

Blank of **1-Naphthaldehyde**

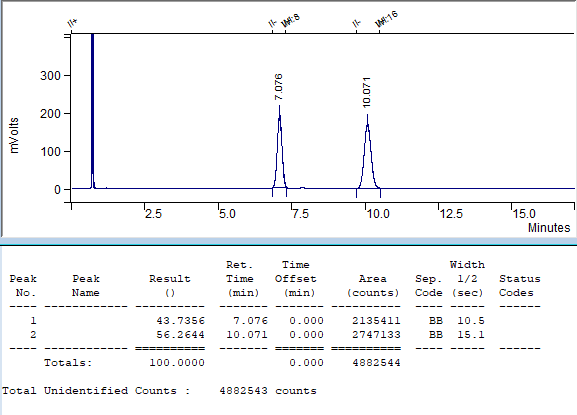

**GC analysis of 2-Naphthaldehyde reduction:**

**
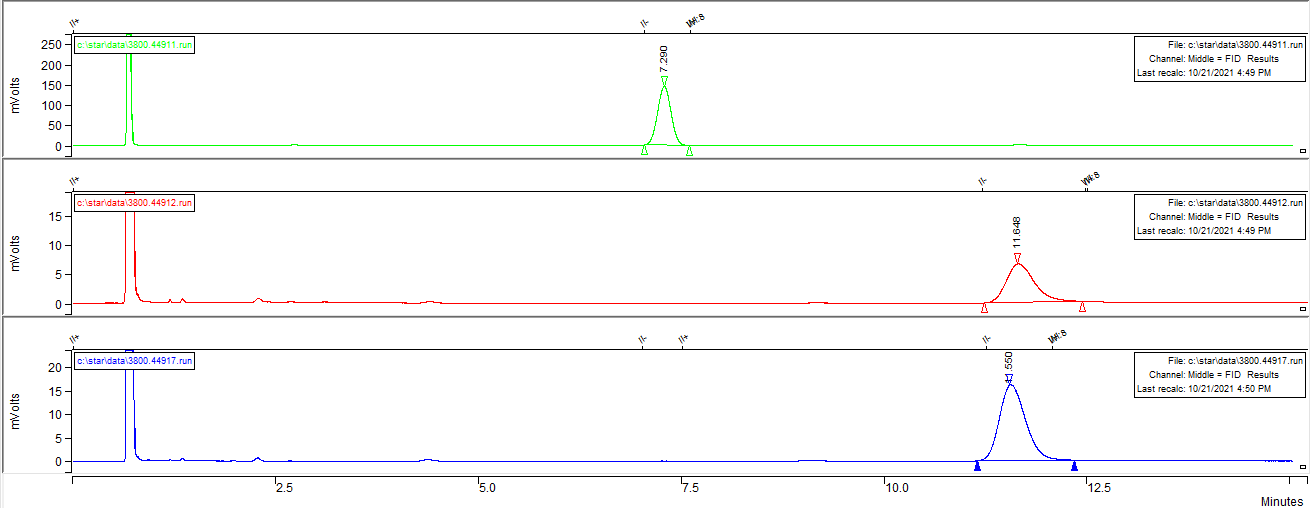
**

Mix of reaction

Blank of **2-Naphthylmethanol**

Blank of **2-Naphthaldehyde**

**
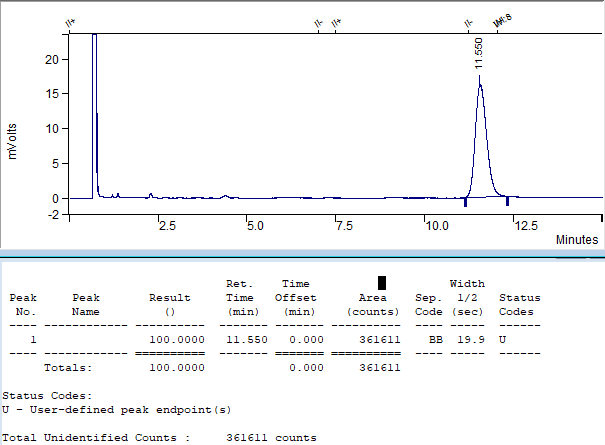
**

**GC analysis of Acetophenone reduction:**

**
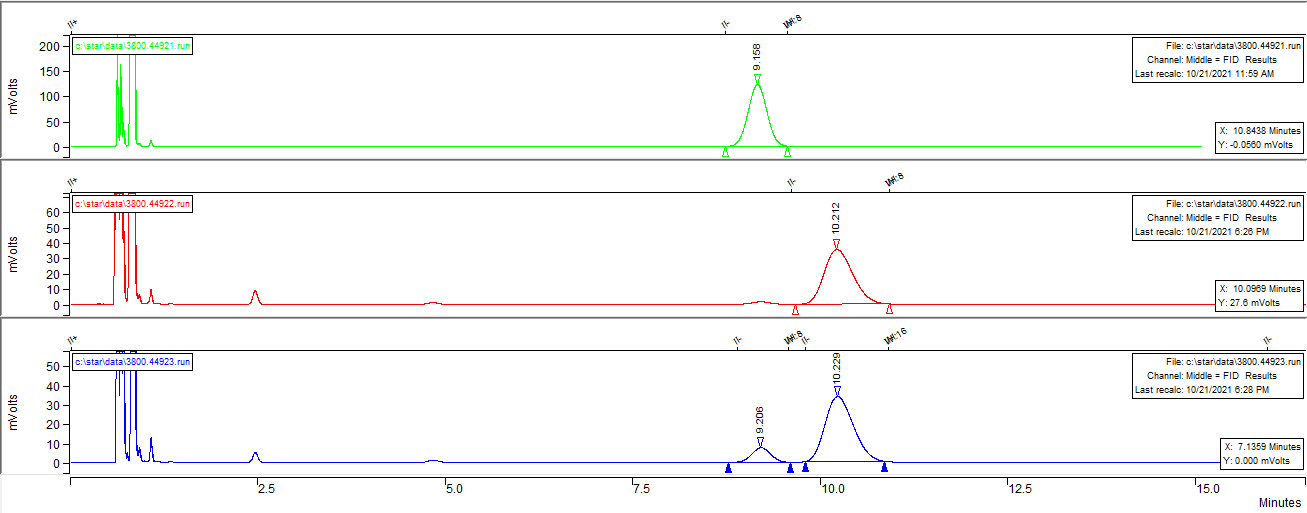
**

Blank of **Acetophenone**

Mix of reaction

Blank of **Phenylethanol**

**
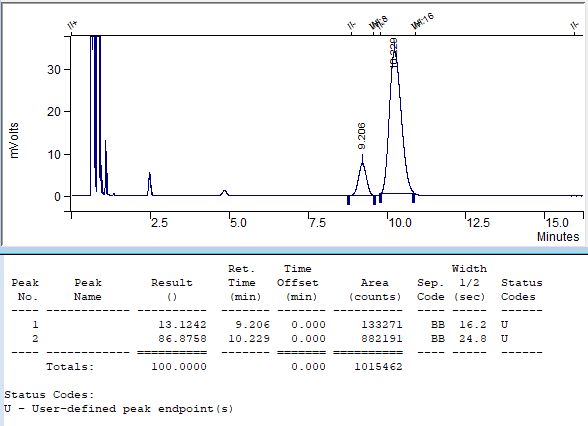
**

**GC analysis of Benzophenone reduction:**

**
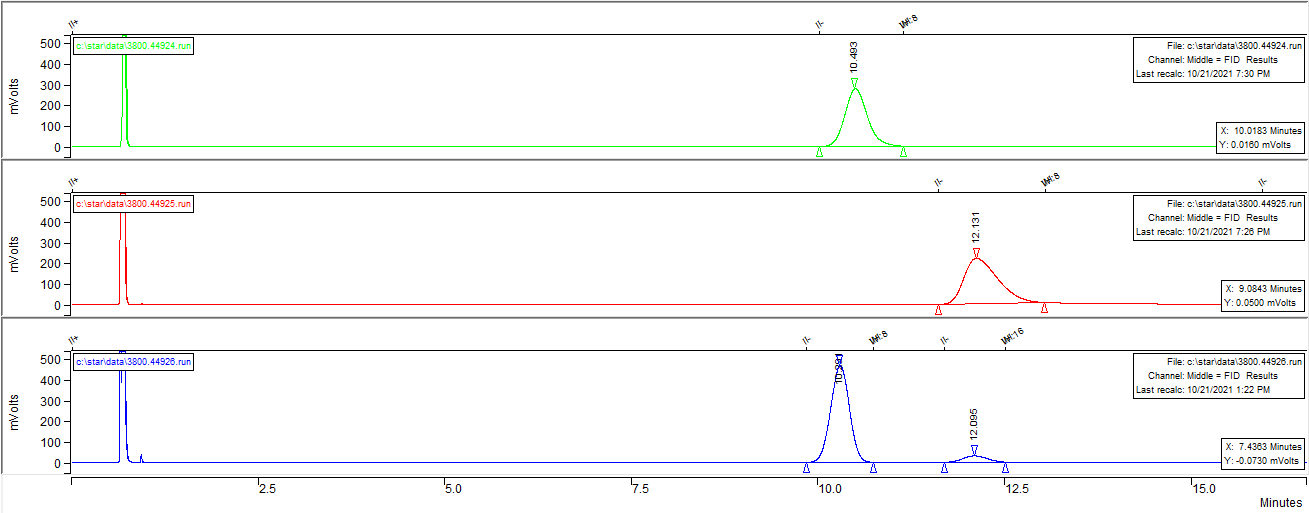
**

Mix of reaction

Blank of **diphenylmethanol**

Blank of **Benzophenone**

**
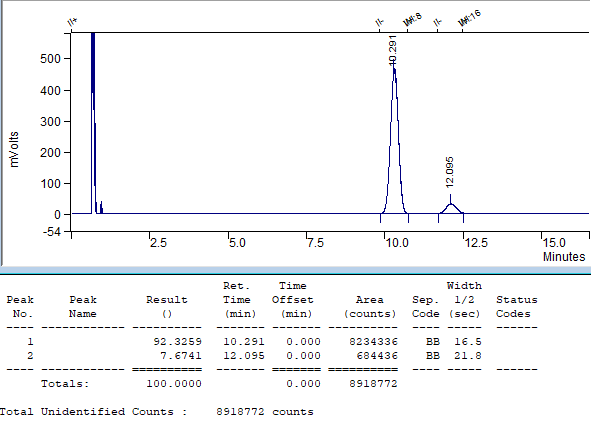
**

**GC analysis of Cyclohexanone reduction:**


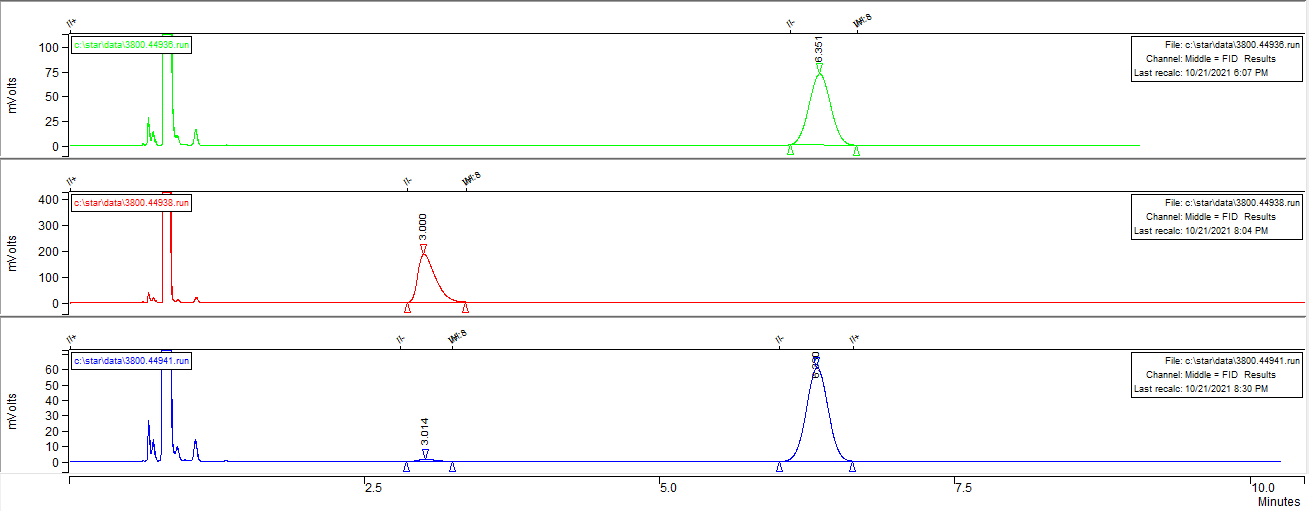

Mix of reaction

Blank of **Cyclohexanol**

Blank of **Cyclohexanone**


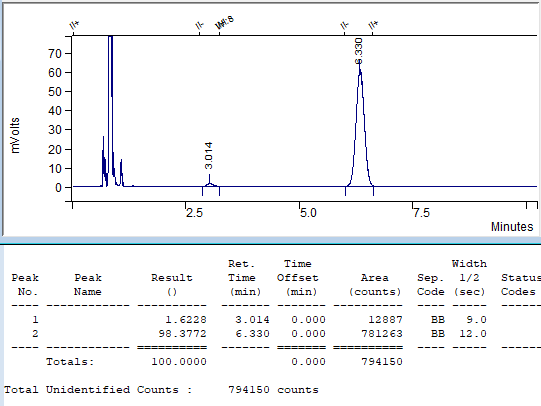

**GC analysis of 1-Hexanal reduction:**


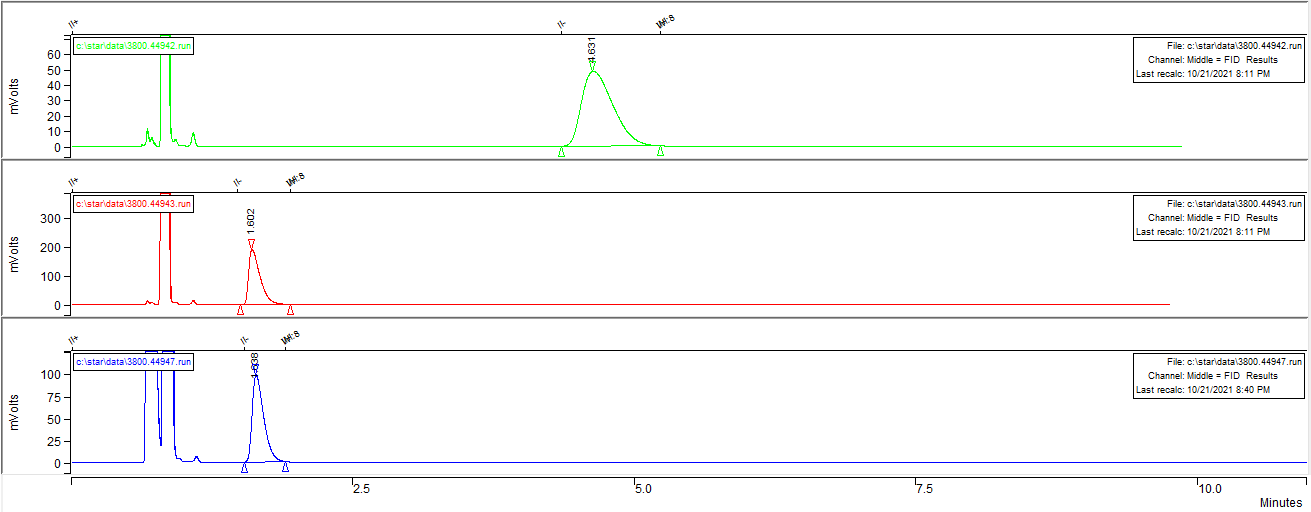


[Mix of reaction](https://en.wikipedia.org/wiki/Heptanal)

[](https://en.wikipedia.org/wiki/Heptanal)

[](https://en.wikipedia.org/wiki/Heptanal)

[Blank of](https://en.wikipedia.org/wiki/Heptanal) **[1-Hexanol](https://en.wikipedia.org/wiki/Heptanal)**

[Blank of](https://en.wikipedia.org/wiki/Heptanal) **[1-Hexanal](https://en.wikipedia.org/wiki/Heptanal)**


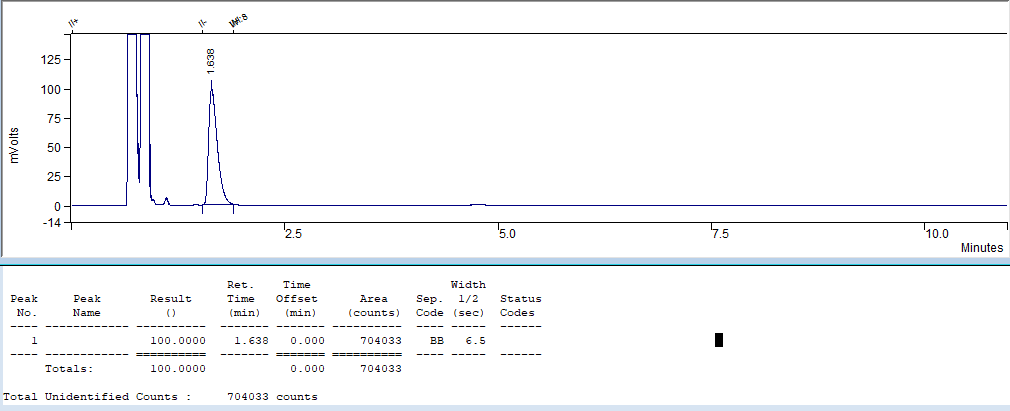

**GC analysis of Styrene reduction:**


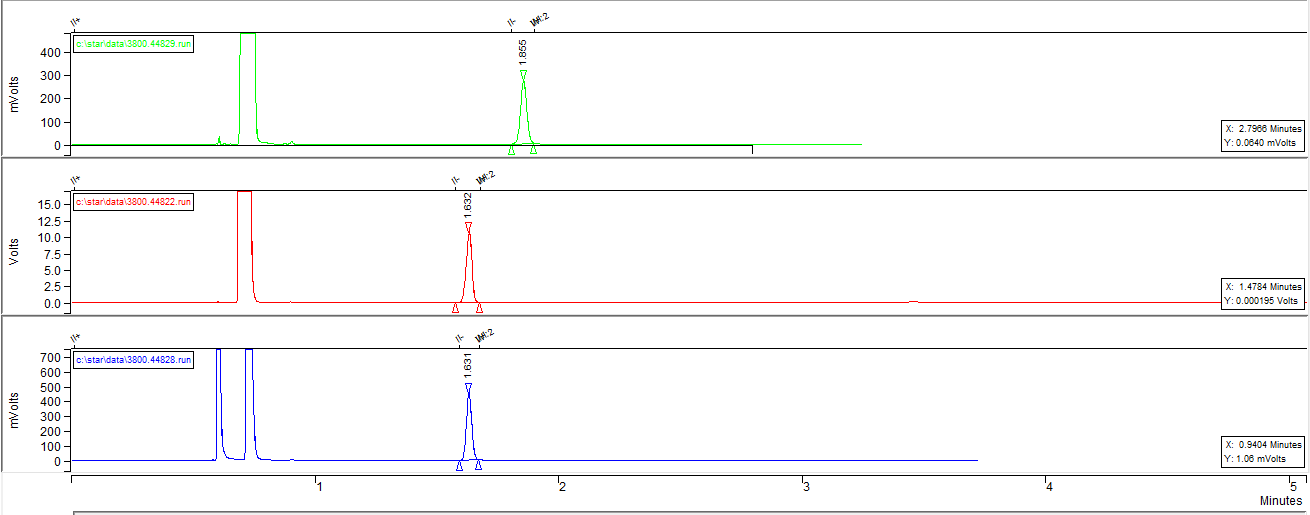


Mix of reaction

Blank of **Styrene**

Blank of **Ethylbenzene**

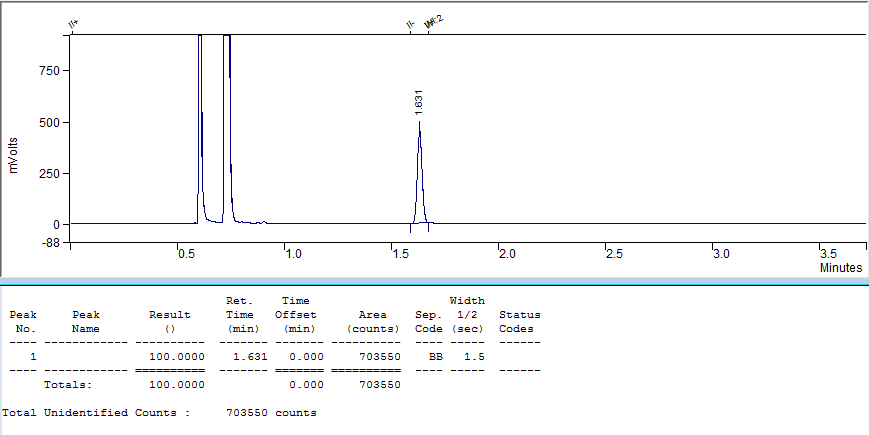

**GC analysis of Phenylacetylene reduction:**

**
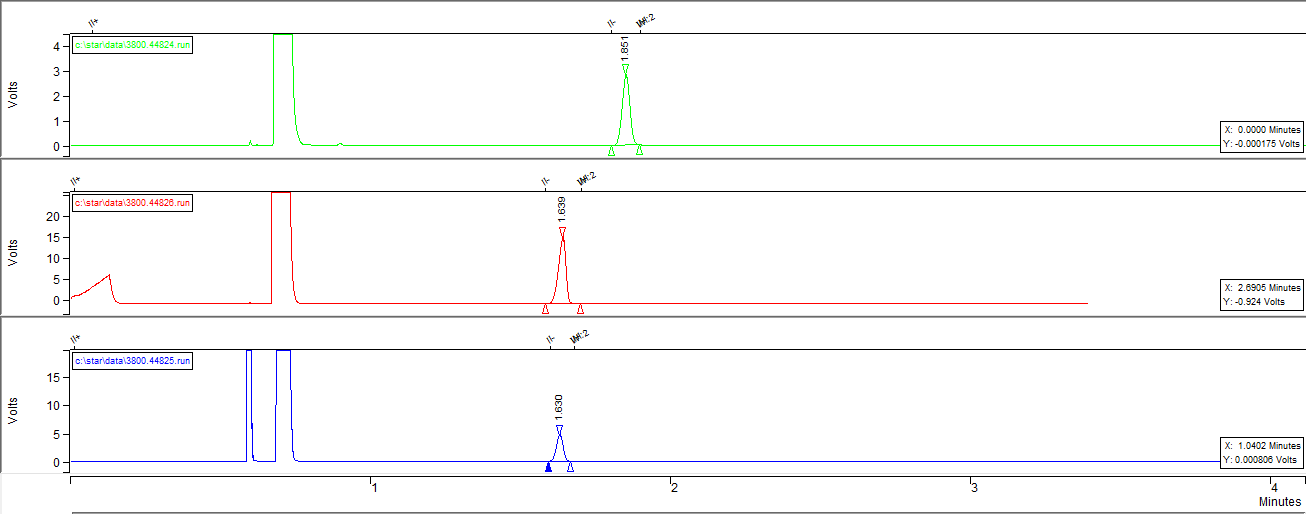
**

Blank of **Ethylbenzene**

Blank of **Phenylacetylene**

Mix of reaction

**
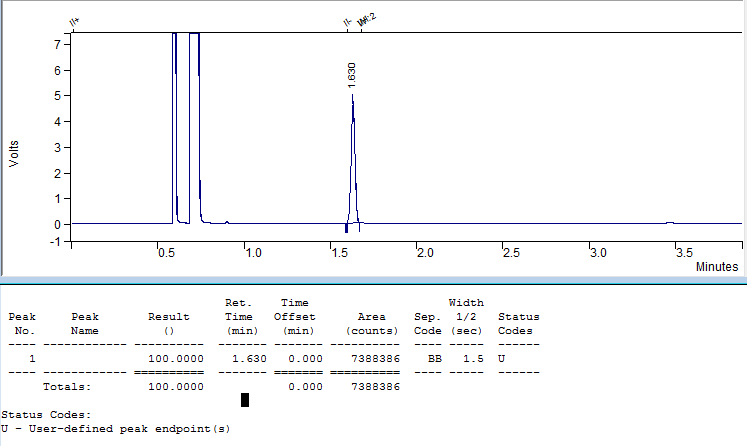
**

**4. Cyclic Voltammetry and Linear Sweep Voltammetry results:**

CV curves (20 mv/s) of Benzaldehyde (0.025 mL) in ethanol and water (1:1) (30 mL) with LiClO_4_ (250 mg) NH_4_PF_6_ (100), Ag /AgCl as a reference and Ni foam as a working and counter electrode.

**LSVs** curves of Benzaldehyde (0.025 mL) in ethanol and water (1:1) (30 mL) with LiClO_4_ (250 mg) NH_4_PF_6_ (100mg), Ag /AgCl as a reference and Ni foam as a working and counter electrode.

CV (20mv/s) of pure Benzaldehyde (0.025 mL) with LiClO_4_ (250 mg) and NH_4_PF_6_ (100mg), Ag wire as a reference and Ni foam as a working and counter electrode.

LSV (20mv/s) of pure Benzaldehyde (0.025 mL) with LiClO_4_ (250 mg) and NH_4_PF_6_ (100mg), Ag wire as a reference and Ni foam as a working and counter electrode.

**Reference:**

1. Tamang SR, Cozzolino AF, Findlater M. Iron catalysed selective reduction of esters to alcohols. ***Org. Biomol. Chem.*,** 2019,**17**, 1834-1838. [10.1039/C8OB02661K](https://doi.org/10.1039/C8OB02661K)

2. Wang Z, Chen X, Liu B, Liu QB, Solan GA, Yang X, Sun WH. Cooperative interplay between a flexible PNN-Ru (ii) complex and a NaBH _4_ additive in the efficient catalytic hydrogenation of esters. ***Catal. Sci. Technol.***, 2017,**7**, 1297-1304. [10.1039/C6CY02413K](https://doi.org/10.1039/C6CY02413K)

3. Yoshida M, Hirahata R, Inoue T, Shimbayashi T, Fujita KI. Iridium-catalyzed transfer hydrogenation of ketones and aldehydes using glucose as a sustainable hydrogen donor. *Catalysts*. 2019;**9(6)**,503. [10.3390/catal9060503](https://doi.org/10.3390/catal9060503)

4. <https://www.chemicalbook.com/SpectrumEN_1777-82-8_1HNMR.htm>
